# Standardizing the management of cardiovascular diseases in the primary health care setting of Pakistan

**DOI:** 10.1186/s12875-025-03143-y

**Published:** 2025-12-26

**Authors:** Alina Pervez, Omar Mahmud, Farhala Baloch, Nashia Ali Rizvi, Saira Bukhari, Bilal Ahmed, Sheema Sadia, Yawer Saeed, Intisar Ahmed, Russell Seth Martins, Mohsin Ali Mustafa, Ainan Arshad, Iman Mushfiq Farooqui, Momina Faisal, Amna Rashid Hanfee, Adil H. Haider

**Affiliations:** 1 Center for Clinical Best Practices, Clinical and Translational Research Incubator, Aga Khan University, Karachi, Pakistan; 2 Medical College, Aga Khan University, Karachi, Pakistan; 3 Section of Cardiology, Department of Medicine, Aga Khan University, PO Box 3500, Stadium Road, Karachi, 74800 Pakistan; 4 Department of Medicine, Aga Khan University, Karachi, Pakistan

**Keywords:** Clinical practice guidelines, Healthcare standardization, Preventive cardiology, GRADE-ADOLOPMENT, Primary health care, Cardiovascular diseases, Ischemic heart disease, Congestive heart failure, Stable angina, Low- and Middle-Income countries

## Abstract

**Background:**

Cardiovascular diseases are increasingly prevalent in Pakistan. The establishment of clinical practice guidelines (CPGs) and primary-care referral pathways can help primary care physicians (PCPs) play a greater role in the management of patients to optimize the delivery of care. In this study, we aimed to develop CPGs and primary-care referral pathways that are specific to Pakistan’s primary-care setting in the context of ischemic heart disease (IHD) and congestive heart failure (CHF).

**Methods:**

The GRADE-ADOLOPMENT approach was utilized, and source guidelines were selected based on relevance, scope, and rigor. Recommendations were scrutinized for adoption, adaptation, or exclusion based on local feasibility and applicability. A structured review process involved internal and external expert reviewers to ensure credibility and relevance. Additionally, contextual clinical referral pathways were crafted to guide PCPs in patient management and specialist referrals.

**Results:**

Through the GRADE-ADOLOPMENT process, contextualized CPGs for managing stable IHD and CHF in Pakistan’s primary care setting were successfully developed. A total of 72 recommendations were excluded from source guidelines due to various reasons, while the remaining recommendations were deemed applicable and adopted into the local CPGs. Clinical referral pathways were drafted to facilitate efficient and resource-saving referrals by PCPs, ensuring appropriate care for patients requiring specialist attention. 4 recommendations were added to the referral pathways to cover gaps in care provision.

**Conclusions:**

This guideline has thus been tailored to suit the local context and support physicians with evidence-based recommendations that are feasible for implementation in the Pakistani setting.

**Supplementary Information:**

The online version contains supplementary material available at 10.1186/s12875-025-03143-y.

## Introduction

Cardiovascular diseases (CVDs) continue to be the leading cause of death worldwide, with ischemic heart disease (IHD) and congestive heart failure (CHF) contributing significantly to morbidity and mortality. Moreover, the prevalence of CVDs continues to increase and has nearly doubled between 1990 and 2019 from 271 million to 573 million cases globally [1]. Unfortunately, estimates indicate that up to 80% of CVD-associated deaths occur in lower and middle-income countries (LMICs) where pervasive risk factors such as tobacco use, hypertension, diabetes, and obesity are compounded by financial and logistical barriers to effective healthcare [1–3]. In Pakistan, a South Asian LMIC, the population is at an increased risk for CVDs and a growing annual CVD-associated death rate of over 500,000 in the coming years has been forecasted [4–6].

Evidence-based clinical practice guidelines (CPGs) are a means to standardize the effective and equitable management of CVDs. In LMICs like Pakistan, CPGs can improve health service delivery by providing practical, context-specific recommendations for widespread adoption. This can include integration of effective, affordable, and locally available drugs and tools to ensure the reliability of care [2]. CPGs may also consider local epidemiology, socio-cultural, and economic factors, as well as the priorities and needs of stakeholders such as native communities. This is especially true of CPGs for CVDs, given that the latter are influenced by a complex interplay of biological, behavioral, social, and environmental risk factors [1].

Within this framework, CPGs can offer structured guidelines and a systematic approach to managing CVDs within the primary care setting to ensure that specialist referral is sought only in cases where necessary [7]. This approach optimizes resource utilization in service of a more sustainable healthcare system. However, LMICs like Pakistan often lack the resources and data needed to develop such guidelines locally [8]. Consequently, healthcare professionals in such regions often turn to guidelines sourced from higher income countries (HICs) from where a significant portion of relevant research originates [9]. Nevertheless, these guidelines do often comment on the management of patients in low-resource settings, and many of the elements from HIC CPGs are universally applicable and based on high quality and generalizable evidence. An effective solution lies in adapting international CPGs to suit the local healthcare landscape while retaining a robust evidence base [8]. By striking a balance between global best practices and the unique healthcare challenges faced by LMICs, these adapted guidelines can provide a practical roadmap for managing CVDs in the primary care setting in a manner that aligns with local realities.

A rigorous and methodical approach to the adaptation of existing guidelines in this manner is the GRADE (Grading of Recommendations, Assessment, Development and Evaluation)-ADOLOPMENT approach [8]. Using existing CPGs as a basis, it enables the adoption (unaltered implementation of recommendations), adaptation (modification of a recommendation from the source guideline following critical appraisal, a systematic review of the local context, and expert panel collaboration), and new development (generation of select recommendations de novo to meet local needs) of recommendations to produce locally relevant guidelines. This adaptive approach ensures that CPGs remain valuable tools in addressing the burden of CVDs while fostering sustainable healthcare practices in LMICs like Pakistan. To ensure the production of high-quality, transparent, and formalized CPGs, the GRADE-ADOLOPMENT process uses evidence to decision (EtD) Table [8]. These allow experts to assess the quality and relevance of source guideline recommendations using both general and context-specific evidence.

We sought to use the GRADE-ADOLOPMENT approach to develop locally contextualized CPGs for the primary care physicians (PCPs) of Pakistan to guide the management of stable IHD and CHF. By employing a transparent and reliable methodology that incorporates the context of the local setting, our aim was to produce credible and practical CPGs that achieve a high local penetrance and better equip Pakistani physicians to grapple with the increasing disease burden of CVDs in the country [10]. We also aimed to develop corresponding clinical referral pathways to be applied in the primary care setting, allowing PCPs to visualize the guideline recommendations and make informed decisions about when to escalate to specialist care.

## Methodology

### Context

The initiative was undertaken collaboratively by the Center for Clinical Best Practices (CCBP) and the Section of Cardiology in the department of Medicine at Aga Khan University Hospital (AKUH) in Pakistan. AKUH is a tertiary care, non-profit, university-hospital in Pakistan. Acknowledging the shortage of specialist cardiologists in the country and the vital role PCPs play in the management and triage of patients with CVDs, the focus of this project was on developing cardiology guidelines for the PCPs in Pakistan.

### Collaborative team

The guideline development team constituted of senior faculty, attending cardiologists (including the Section Head of Cardiology at AKUH), and CCBP personnel skilled in guideline development. The GRADE-USA working group provided training for the CCBP team, faculty, and staff involved in the GRADE-ADOLOPMENT process.

### Source guidelines selection

The choice of CVDs to include was based on consensus of the specialist clinicians at AKUH. Subsequently, a collection of source guidelines was compiled following a literature review driven by searches of the Medline and Scopus databases (for articles published between 2010 to July 2022). Cardiologists in the ADOLOPMENT committee considered their experience with the use of guidelines from professional societies in HICs such as The American College of Cardiology and European Society of Cardiology. The selection process was based on criteria including the scope of the guideline, credibility of the professional organizations developing or endorsing the guidelines, local relevance, and rigor of the methods used to develop the CPGs. The aim of the selection process was to identify source guidelines with broad scope that considered all aspects of the modern management of IHD and CHF based on the highest quality of evidence. This was to ensure that the subsequent ADOLOPMENT process could generate locally applicable recommendations for PCPs using high quality foundational resources, even if these were not easy to translate directly to the Pakistani context. The source guidelines selected were thus from the American College of Cardiology and American Heart Association as follows:

- I. Guideline for the Diagnosis and Management of Patients with Stable Ischemic Heart Disease - American College of Cardiology Foundation and the American Heart Association, 2012 [11].
- II. Focused Update of the Guideline for the Diagnosis and Management of Patients with Stable Ischemic Heart Disease - the American College of Cardiology Foundation and the American Heart Association, 2014 [12].
- III. AHA/ACC/HFSA Guideline for the Management of Heart Failure: A Report of the American College of Cardiology/American Heart Association Joint Committee on Clinical Practice Guidelines, 2022 [13].

### Adolopment process

**Fig. 1:**
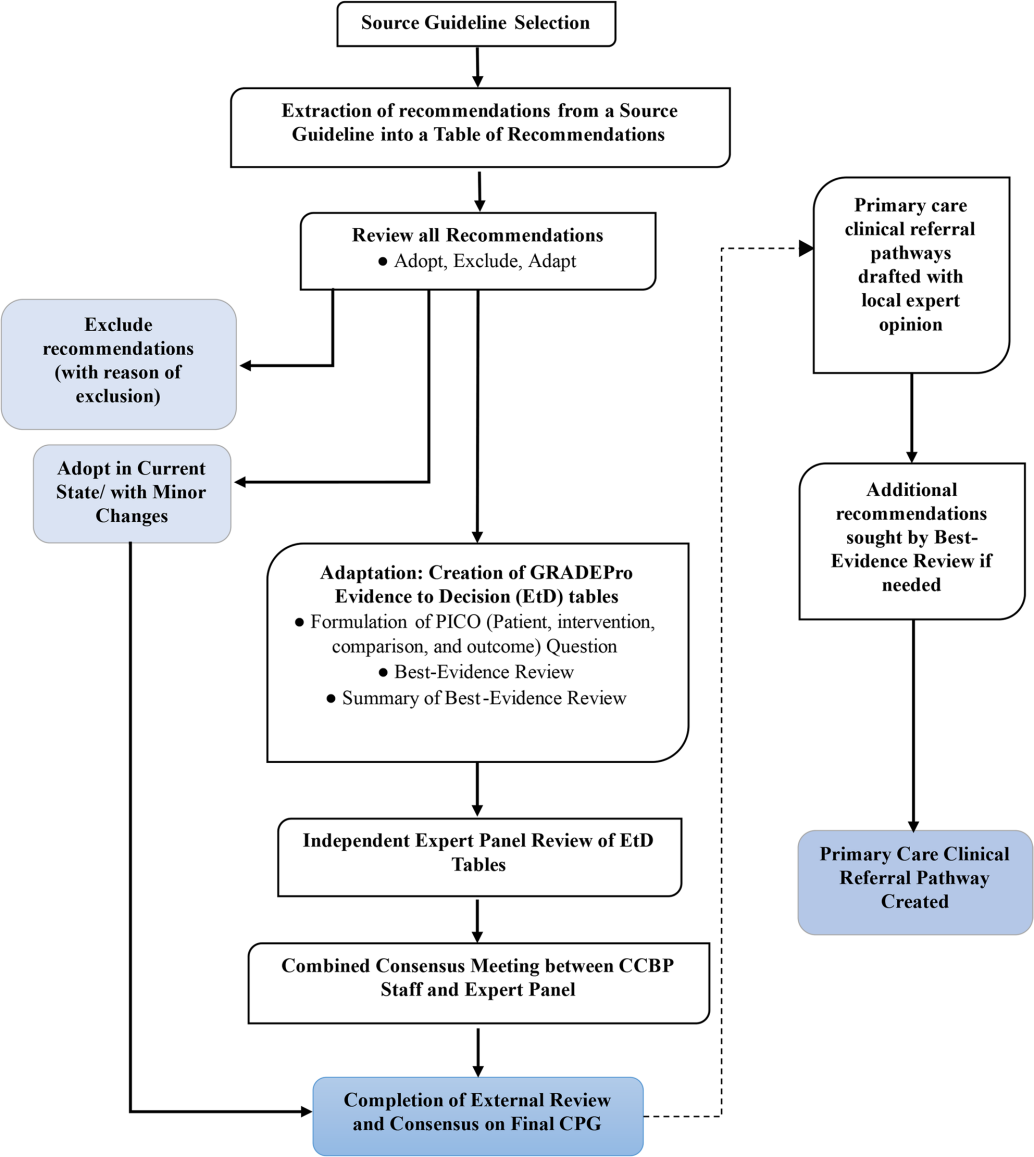
Guideline and Clinical Referral Pathway Creation Process

The ADOLOPMENT process was used to create contextualized guidelines (Fig. 1). The Table of Recommendations (ToR) was formulated via extraction of all recommendations from the source guidelines. Senior cardiologists individually adjudicated each recommendation with designations of “Adopt,” “Adapt,” or “Exclude.” Final determinations were made by consensus following discussion and guidance from the Section Head. Recommendations marked “Adopt” retained their original form, while “Exclude” recommendations were omitted, particularly if they pertained to inpatient care, recommended therapy being unavailable, or proved irrelevant within the Pakistani context. For recommendations labeled “Adapt,” an additional process through the GRADEPro process was to be embarked upon, ensuring a thorough review and refinement (Supplementary Material).

The adoloped guidelines subsequently underwent a rigorous review process, which included internal reviewers comprising healthcare experts from AKU, as well as external reviewers who were healthcare experts from Pakistan not affiliated with the institution. These reviewers were given access to the CPGs and were allowed to evaluate them independently. All suggested changes were then returned to the ADOLOPMENT team and considered for incorporation by the group, with revisions made on the basis of consensus.

### Referral pathways

Led by the cardiologists in the team, management and referral algorithms were produced for use by PCPs. The algorithms were supported by recommendations from the contextualized CPGs and related to the processes of screening, diagnosis, intervention, and timely specialist referral. Where existing guidelines did not provide sufficient recommendations, an effort was made to identify additional guidance to fill these gaps via a best evidence review process.

### CPG dissemination

We have devised a comprehensive dissemination strategy to promote awareness and the adoption of our guidelines. This strategy encompasses several channels, including peer-reviewed journal publication and the release of “The AKU Manual of Therapeutics” in book format. Furthermore, we plan to make these guidelines readily available as a digital resource via the AKU Web app. Additionally, we will develop digital lectures based on the guidelines to facilitate broader access to the content. We intend to present these guidelines at both national and international conferences. Our overarching goal in disseminating these guidelines widely is to ensure that healthcare professionals across our resource-limited country can readily access and benefit from this resource.

### Chronology of progress

The development of the guidelines and clinical pathways spanned the period from June 2022 to January 2024:

- Source CPGs Selection: June-August 2022
- Table of Recommendations Creation: September-November 2022
- Table of Recommendations Review: December 2022- March 2023
- Final CPG Crafting: April 2023
- Drafting Primary Care Clinical Referral Algorithm: June-August 2023
- Seeking Additional Recommendations: September-October 2023
- Finalizing Primary Care Clinical Referral Algorithm: December 2023 -January 2024

## Results

As a result of the GRADE-ADOLOPMENT process, we successfully created two contextualized CPGs for the management of stable IHD and CHF in the primary care setting of Pakistan. 72 recommendations were excluded from the source guidelines; the reasons varied from local unavailability of services and medication to recommendations pertaining to in-patient care (Supplementary Table 3). All other recommendations were deemed acceptable in the local setting and were adopted as is into the local CPGs (Supplementary Material).

**Fig. 2:**
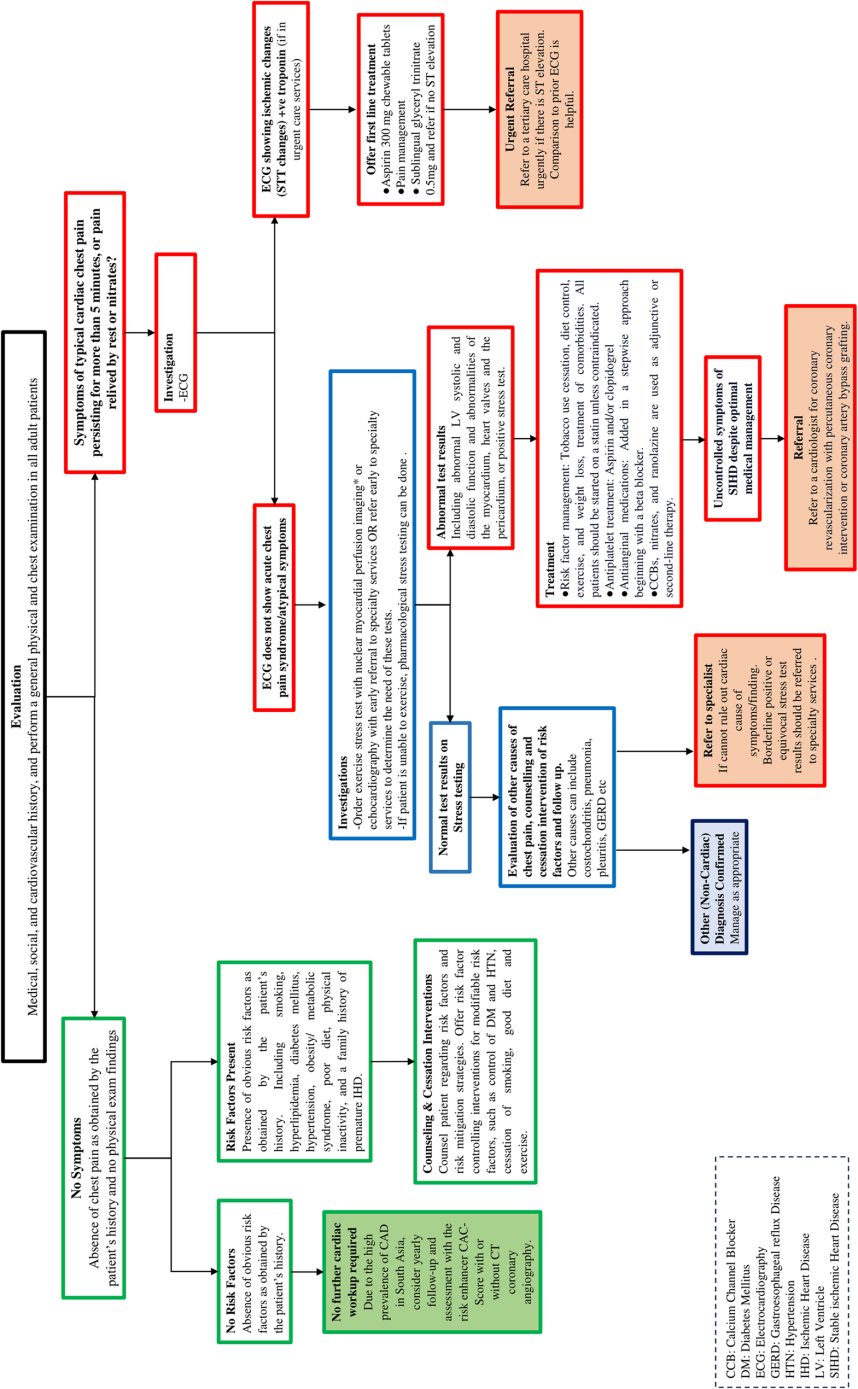
(Disclaimer: These algorithms are for use by Primary Care Providers in Pakistan managing typical patients with ischemic heart disease. Providers are encouraged to exercise clinical judgement, consider referral for patients with complex disease, and refer to specific guidelines that deal with ischemic heart disease with complex or atypical features when managing their patients)

**Fig. 3:**
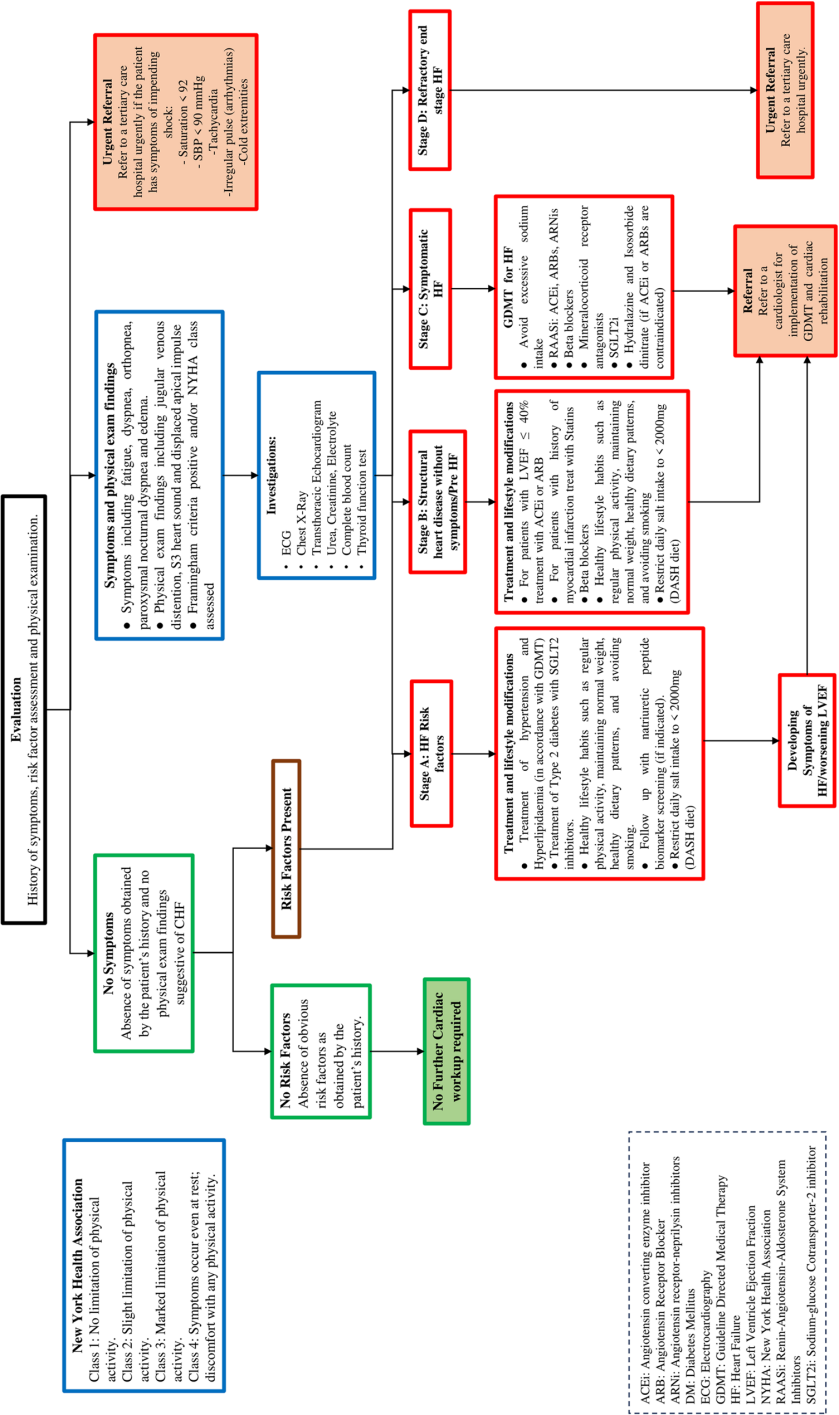
Primary Care Referral Pathways for Congestive Heart Failure. (Disclaimer: These algorithms are for use by Primary Care Providers in Pakistan managing typical patients with congestive heart failure, primarily with reduced ejection fractions where LVEF is less than 40%. Providers are encouraged to exercise clinical judgement, consider referral for patients with complex disease, and refer to specific guidelines that deal with Heart Failure due to specific etiologies when managing their patients. Management of patients with HFpEF should be adjusted based on the differences between therapeutics with proven benefit in HFrEF versus HFpEF)

Primary care clinical referral pathways were drafted from the contextualized CPG. Recommendations were added to the algorithm from reputable guidelines where a gap was found in the provision of patient care (Figs. 2 and 3). A total of four recommendations were added to referral pathways (Table 1). Providers should note that these algorithms provide guidance for treatment and referral by PCPs in Pakistan managing typical patients with IHD or CHF (primarily patients with an LVEF of < 40%. PCPs may adapt this guidance to patients with preserved ejection fractions using clinical judgement and recognizing the different evidence based therapeutics available for the management of HFpEF). Providers are encouraged to exercise clinical judgement, consider referral for patients with complex disease, and refer to specific guidelines that deal with Heart Failure due to specific etiologies when managing their patients.

**Table 1:** Recommendations added in the clinical referral pathways for the evaluation and management of congestive heart failure and stable ischemic heart disease

Action	Recommendation	Source for inclusion
Congestive Heart Failure		
Added	Management of shock requires tertiary care and/or in-patient care if possible. Therefore, referral by GPs of patients on an urgent basis to a tertiary care center is recommended if signs and symptoms portend impending shock (Sat. <92, SBP < 90mmHg, Tachycardia, irregular pulse/arrhythmia, cold extremities).	UpToDate, MSF Medical Guidelines[14, 15]
Stable Ischemic Heart Disease		
Added	Given the high regional prevalence of CAD, consider measuring risk enhancers e.g. CAC score +/- CT CA in the absence of signs and symptoms or risk factors and an annual follow-up with a primary care physician	UpToDate[16, 17]
Added	SIHD - Negative initial ECG does not rule out ACS/myocardial ischemia; if ECG is negative after indicated testing, • refer to a specialist to evaluate the need for further testing. or • order subsequent tests and refer patients with borderline positive or equivocal stress test results to specialty services	UpToDate[18–20]
Added	SIHD - Comparison to prior ECG may be helpful at the time of referral of patients with ST-elevation on ECG as the ECG criteria for MI refer to ‘new’ changes.	UpToDate[19]

*ACS* acute coronary syndrome, *CAC* coronary artery calcification, *CAD* coronary artery disease, *CHF* congestive heart failure, *CT. CA* computed tomography coronary angiography, *ECG* electrocardiogram, *GP* general practitioner, *MI* myocardial infarction, *Sat.* saturation, *SBP* systolic blood pressure

## Discussion

In this article, we report evidence-based CPGs for the outpatient management of adult stable IHD and CHF by PCPs in Pakistan. Two source guidelines and one focused update were used in the GRADE-ADOLOPMENT process. Seventy-two recommendations were evaluated and determined to be unsuitable for inclusion in the CPGs produced. Moreover, an associated referral pathway was produced to accompany each set of guidelines to facilitate efficient triage and referral to specialist care when necessary. Our implementation of this rigorous and transparent methodology ensured that the CPGs produced are credible and can be implemented reliably and effectively in the Pakistani context, while also serving as a foundation for future iteration and as a guide for subsequent research.

Pakistan’s rapidly expanding populace of well over 200 million people is exposed to a unique combination of risk factors for CVDs, such as high rates of diabetes mellitus, hypertension, smoking, and genetic risk associated with prevalent consanguinity [21, 22]. Recent estimates suggest that the local age-standardized incidence of CVDs is as high as 918 per 100,000, over 1.3 times greater than the global average [23]. These and other data suggest that lower thresholds for CVD screening may be prudent in our setting than elsewhere. Our guidelines acknowledge this by focusing on the primary care setting and equipping PCPs with the decision-support needed to adequately address CVDs across various stages. Moreover, we have addressed use of clinical, laboratory, and imaging tools such as cardiac biomarkers including natriuretic peptide assays and coronary artery calcium scores to screen and stratify patients with possible subclinical CHF and IHD. However, the timely identification of patients is only one of the challenges to optimizing cardiovascular care for Pakistanis, who are served by as few as 1.09 doctors and 0.59 allied health workers per 1000 people and just 1147 cardiologists with certified fellowship training who largely practice in major urban centers [22]. Evidence suggests that effectively curtailing the effects of CVDs in Pakistan will require community-based approaches that emphasize prevention strategies, regular follow-up, and early intervention in a manner that patients are willing to adhere to [24]. Thus, the approach to tackling CVDs in Pakistan must be of sufficiently large scale, tailored to local conditions, and feasible despite the dearth of specialists.

Between these conditions and the chronic nature of CVDs – where the long course of clinical management is influenced by patient lifestyle, sociocultural norms, and the availability and affordability of diagnostic and therapeutic options – PCPs must be empowered to play a leading role in patient care and referral. However, local guidelines for the management of CVDs are limited in scope, apply to narrowly defined patient populations, and do not report methodologies used to develop recommendations [25, 26]. Moreover, it was previously unclear how PCPs might apply foreign guidelines as their suitability for the Pakistani healthcare system had not been systematically appraised. Having provided and highlighted evidence-based CPGs that incorporate only relevant and accessible options to local providers, our results may facilitate the critical role of these physicians in providing quality care to Pakistanis suffering from CVDs.

It is equally essential to ensure that specialist care is directed towards appropriately selected patients who suffer from complex or advanced stages of CVDs to prevent debilitation or death. Evidence has shown that the dissemination of guidelines with structured referral pathways can improve the effectiveness and efficiency of outpatient referrals to specialists [27]. Consequently, our results include such referral pathways that clearly define the appropriate reassurance, counseling, management, or referral for patients under the care of primary care physicians.

An additional benefit of our implementation of the GRADE-ADOLOPMENT approach is its utility in highlighting scope for future research. Niches of clinical practice or scenarios that are inadequately addressed by the adoloped guidelines may represent areas that require new or adapted recommendations. These might be prioritized for future research when limited local resources are to be allocated. Thus, in addition to producing credible CPGs that local physicians may be more willing to adopt, our work may help to guide the efficient allocation of resources and personnel for future research in Pakistan as well as in other settings [10, 28].

Our recommendations draw attention to important challenges faced by primary care physicians in Pakistan. Several recommendations were omitted due to the local unavailability of medical devices, procedures, or drugs. For example, evidence suggests that the use of wearable implantable hemodynamic monitoring devices may predict or even prevent hospitalization of some patients with heart failure, such as those on a trajectory leading toward decompensation [29, 30]. However, these devices are not realistic options for PCPs to consider due to a lack of infrastructure and supply. Similarly, drugs like potassium binders have established clinical utility in the management of CHF but are also not available in Pakistan [31]. Our guidelines also do not distinguish between the use of angiotensin-receptor-neprilysin inhibitors (ARNIs) versus conventional angiotensin converting enzyme inhibitors (ACEIs) or angiotensin receptor blockers (ARBs). Although guidelines in HICs have increasingly supported the use of ARNIs, these are more expensive than widely available generic forms of ACEIs and ARBs [13]. Furthermore, the latter are drugs that have been in clinical use for significantly longer and PCPs in Pakistan are more experienced with their use, which can be particularly important when managing frail, geriatric, or multimorbid patients who are more likely to experience drug side effects. Our guidelines recognize the importance of cost, availability, and experience to PCPs when prescribing in complex patients such as those with CHF [32]. Ultimately, Pakistan is a developing country where some gap between advances in healthcare and the feasibility of local implementation is inevitable, and clinicians must work within these bounds. Compounding this challenge is the fact that most healthcare in Pakistan is financed out-of-pocket by uninsured patients, resulting in unacceptably high costs to patients [31, 32]. It is paramount that PCPs provide care that is effective but also without the risk of financial catastrophe or serious disruption to daily life whenever possible. Infeasible recommendations, such as those involving the drug Tafamidis (which costs up to 225,000 USD per year) were thus excluded from the CPGs [33]. Having ensured that all recommendations made by these CPGs are relatively feasible in local clinical settings ensures their practical utility, which is a strength of our study and a benefit of utilizing the GRADE-ADOLOPMENT approach. Physicians may also be confident that the referral pathways produced account for, to the extent possible, limitations inherent to the Pakistani health system.

Our work does have several limitations that are to be acknowledged. The appraisal of source guidelines during the ADOLOPMENT process does involve decisions made on the basis of expert consensus, which inevitably results in some degree of subjectivity. Furthermore, key stakeholders who will be involved in the implementation and use of these CPGs, such as PCPs, nurses, other allied health professionals, government officials, and patients/community members were not equally involved in the ADOLOPMENT process. Furthermore, all experts were from our own institution, and those at other academic centers and hospitals or in settings such as private practices were not consulted during initial development. However, the decision to restrict involvement of multiple parties during ADOLOPMENT was to avoid delays due to logistical constraints, conflicts of interest, political influence, and the absence of direct incentives. Moreover, external reviewers played a significant role in later stages of the process. These limitations represent difficulties associated with the practical implementation of the GRADE-ADOLOPMENT process, particularly in settings where such interdisciplinary academic collaborations may not be routine or where resources are limited, as is the case in Pakistan. However, due to our team’s extensive experience in developing CPGs for use by Pakistani primary care physicians, we are confident that the requirements and preferences of these stakeholders will have been comprehensively incorporated into the ADOLOPMENT process. Moreover, these CPGs may act as a foundation on which insights provided by these stakeholders as well as the findings of future research conducted de novo in Pakistan may be used to make additions and improvements, such as new or adapted guidelines to further improve the suitability of these recommendations for patient care in the local context.

### Comprehensive plan for training, implementation, and sustainability

In our upcoming initiatives, we aim to enhance training, implementation, and sustainability of our clinical practice guidelines. We plan to conduct video lectures, led by the Cardiology faculty and our expanded study team, to emphasize the rationale and practical application of recommendations for primary care providers. Specialized training workshops will equip them with the necessary skills. Additionally, we will create digital and printed educational resources to support guideline implementation. Our goal is to distribute the AKU Manual of Therapeutics to trainee physicians across the country, ensuring widespread benefits for patients. For implementation, we will employ a robust strategy, involving continuous monitoring, feedback collection from healthcare providers and patients, and adaptation to overcome challenges. Furthermore, the guidelines will undergo periodic updates to stay aligned with emerging evidence and evolving healthcare trends, with the input of advisory boards and committees.

## Conclusion

This paper reports CPGs Adoloped from evidence-based source guidelines for use by Pakistani primary care physicians in the management of CVDs. GRADE-ADOLOPMENT approach is a transparent and rigorous methodology used to develop these credible, relevant and applicable guidelines, that the physicians can easily implement in their clinical practice. Furthermore, two structured referral pathways for use when managing patients with stable IHD or CHF have been provided to facilitate efficient and resource-saving referral by primary care doctors to ensure appropriate care for patients requiring specialist attention. The tailored guidelines address the unique social, and cultural challenges and resource limitations in the local context. By empowering primary care physicians with these CPGs, we aim to enhance the quality of care for patients with CVDs and alleviate the burden on specialist services. Future research may be warranted to focus on areas where gaps were identified and build upon these CPGs with evidence generated de novo in scenarios where the availability of devices or pharmacological agents change and based on experiences of and feedback raised by stakeholders, such as the primary care physicians who adopt these guidelines. 

## Supplementary Information

Supplementary Material 1.

## Data Availability

All data used in the study is provided in the manuscript and the supplementary material.

## Abbreviations

AF: Atrial fibrillation
ACS: Acute coronary syndrome
ACEi: Angiotensin-converting enzyme inhibitor
AKUH: Aga Khan University Hospital
ARB: Angiotensin receptor blocker
ARNi: Angiotensin receptor neprilysin inhibitor
CT: Computed tomography
CAD: Coronary artery disease
CCBP: Centre for clinical best practices
HF: Heart failure
CHF: Congestive heart failure
HFpEF: Heart failure with preserved ejection fraction
HFrEF: Heart failure with reduced ejection fraction
CPG: Clinical practice guidelines
CVD: Cardiovascular disease
CPET: Cardiopulmonary exercise testing
CMR: Cardiac magnetic resonance imaging
CRT: Cardiac resynchronization therapy
DCM: Dilated cardiomyopathy
DOAC: Direct acting oral anticoagulant
ECG: Electrocardiogram
EF: Ejection fraction
EtD: Evidence to decision
GDMT: Guideline directed medical therapy
ICD: Implantable cardioverter-defibrillator
IHD: Ischemic heart disease
LVAD: Left ventricular assist device
LBBB: Left bundle branch block
LVEF: Left ventricular ejection fraction
MRI: Magnetic resonance imaging
MRA: Mineralocorticoid receptor antagonist
MI: Myocardial infarction
NYHA: New York Heart Association
NT-pro BNP: N-terminus prohormone brain natriuretic peptide
NSAID: Non-steroidal anti-inflammatory drug
PCP: Primary care physician
PET: Positron emission tomography
QoL: Quality of life
TTE: Transthoracic echocardiogram
TIA: Transient ischemic attack
ToR: Table of recommendations
SCD: Sudden cardiac death
