# Supplementary material for: Standardizing the management of cardiovascular diseases in the primary health care setting of Pakistan
Source: BMC Prim Care. 2025 Dec 26;27:25. doi: 10.1186/s12875-025-03143-y (PMC12849105; doi:10.1186/s12875-025-03143-y)
Supplement: Supplementary file 1 — Supplementary Material 1. [file 12875_2025_3143_MOESM1_ESM.docx]

**Supplementary Material**

1. *GRADE-ADOLOPMENT process for adaptation using Evidence to Decision (EtD) tables.*
2. *S1 Table: Criteria evaluated in the Evidence to Decision (EtD) tables.*
3. *S2 Table: Sample of Evidence to Decision (EtD) table*
4. *S3 Table: List of excluded Recommendations*
5. *Table of Recommendations for the management of Congestive Heart Failure*
6. *Table of Recommendations for the management of Stable Ischemic Heart Disease*

**GRADEPro Evidence to Decision Framework**

A web tool called GRADEPro was used to create, organize, and share collections of evidence summaries (1). The CCBP staff members who worked on this project have finished a training course to become proficient in using GRADEPro for the GRADE-ADOLOPMENT procedure. For any suggestions tagged as "Adapt," GRADEPro will be utilized to create Evidence to Decision (EtD) tables that will be used to obtain a consensus.

The EtD table is a structure that enables members of an expert panel to make suggestions or choices about medical care based on compiled evidence. “*Should the Intervention/Suggested Change be favoured over the Comparison/Current Standard of Practice?”* is the question that serves as the starting point for the creation of EtD tables. Using the 12 criteria listed in **Supplementary Table 1**, an expert panel assesses the benefits and drawbacks of the suggested adjustment.

Each criterion is supported with evidence gathered through a best evidence review process, to provide local context for the pros and cons of the recommendation. The CCBP team summarized the newly gathered evidence for each criterion in the “*Research Evidence*” and “*Additional Considerations*” columns. The GRADE-USA working group is closely involved in the creation of the EtD tables.

**Best-Evidence Review**

A best-evidence review was performed to find research evidence against which the recommendation can be evaluated across the 12 criteria. The best-evidence assessment was performed separately for each of the 12 criteria and included a mini-systematic review and collection of supporting evidence.

- *Mini-Systematic Review*: A mini-systematic review follows the same general process as a full systematic review. However, it applies arbitrary selection criteria (such as publications’ geographical area) or a limit on the number of databases searched (2). In our mini-systematic review, PubMed and Google Scholar will be queried using a search string generated using keywords from the recommendation in question. Only articles reporting data relevant to Pakistan was selected in order to maintain a local focus.

Two CCBP members independently screened titles and abstracts of articles sourced from PubMed and Google, and only articles that reported Pakistan-specific information were evaluated in full text for final inclusion. Since the source guide itself was created based on a systematic review process, a careful review of the entire text of the bibliography in the source document was also performed. The two CCBP members then extracted pertinent evidence from the final list of articles and summarized it in the EtD against the respective criteria.

- *Supporting evidence*: Information on the costs of various investigations and treatments, as well as the availability of diagnostic and management facilities, were collected as needed from several local hospitals, facilities health, and pharmacy through telephone inquiries and websites.

**Expert Panel Review**

An expert panel of OBGYN faculty from AKU will be invited by the Department of Obstetrics and Gynaecology to review the completed EtD table for each recommendation and provide their judgement for each criterion. This judgment is in the form of a single selection from multiple response options. If, for any criteria, an expert requires additional evidence, they will be instructed to inform the CCBP team. An effort is made to source the requisite information, which, if found, will be shared with all the panel members. Experts’ judgements will be sought in an anonymous and confidential manner, with the GRADEPro software allowing reviewers to select options and provide feedback without their identity known to fellow experts or the CCBP team. A sample of a GRADEPro EtD is shown as **Supplementary** **Table 2**.

**Final Recommendation Revisions & Synthesis**

After determining the relative merits of each item, the CCBP staff will convene a meeting with the expert panel to discuss the anonymized EtD tables and come to an agreement on whether and how any adjustments to the aforementioned recommendations should be made. Additionally shown and discussed were the referral care paths for each skin disease, and any adjustments that were required were made. The Section Head was given the final version of the developed CPGs and the referral care pathways, after which the suggestions were included in the local guideline along with a summary of the consensus decision.

| **Supplementary Table 1: Criteria evaluated in the Evidence to Decision (EtD) tables** | | |
| --- | --- | --- |
| **Criteria** | **Description** | **Interpretation** |
| **Problem** | The magnitude of a problem, as measured by its prevalence and severity in a local context | The more serious or urgent a problem is, the more likely that the option that better addresses the problem receives a strong recommendation. |
| **Desirable Effects** | The magnitude of desirable effects is judged by considering the importance of the outcome and the size of the desirable effects (likelihood of experiencing a benefit or degree of benefits an individual experiences). | An option with greater desirable effects is more likely to gain a strong recommendation. |
| **Undesirable Effects** | The magnitude of undesirable effects is similarly judged by considering the importance of the outcome and the size of the undesirable effects. | An option with fewer undesirable effects is more likely to gain a strong recommendation. |
| **Certainty of Evidence** | Determined by likelihood that the research provides valid evidence regarding the effect of the option on all critical outcomes. | Evidence with higher certainty lends to a strong recommendation. |
| **Values** | Magnitude of value is judged by the variability or uncertainty of weightage placed upon the outcome by individuals. | Less variability/uncertainty of value leads to a strong recommendation. |
| **Balance of Effects** | The balance of effects is judged by considering the value individuals place upon the main outcomes, the degree of desirable and undesirable effects, and the certainty of those estimates. | The overall balance of effects can be judged as either favoring the intervention or comparison. |
| **Resources Required** | An estimate of the cost of the difference in resource use between the intervention and comparison. | An option with large savings is more likely to receive a strong recommendation. |
| **Certainty of Evidence of Required Resources** | It is determined by the likelihood that the research provides valid evidence of cost differences between the intervention and comparison. | Evidence with higher certainty lends to a strong recommendation. |
| **Cost-Effectiveness** | Determines cost-effectiveness by considering uncertainty about or variability in costs or net benefit, sensitivity analyses, and the reliability and applicability of the economic evaluation. | An option that is more cost-effective is more likely to receive a strong recommendation |
| **Equity** | Likelihood of differences in the relative effectiveness of the intervention for disadvantaged subgroups that influence the absolute effectiveness of the intervention. | An option with a greater likelihood to favor equity is more likely to receive a strong recommendation. |
| **Acceptability** | Likelihood of key stakeholders to accept the distribution of benefits, harms, costs, and ethical concerns associated with the intervention, over an extended period. | An option more acceptable to most stakeholders is more likely to receive a strong recommendation. |
| **Feasibility** | Practicality of sustained use of the intervention. | An option more feasible to most stakeholders is more likely to receive a strong recommendation. |

| **Supplementary Table 2: Evidence to Decision (EtD) table Sample** | | | |
| --- | --- | --- | --- |
| **Question**: Should *Intervention/Suggested Change* be favored over *Comparison/Current Standard of Practice*? | | | |
| **Criteria** | **Research Evidence** | **Additional Considerations** | **Judgment** |
| **Problem:** Is the problem a priority? |  |  | - No - Probably No - Probably Yes - Yes - Varies - Don’t Know |
| **Desirable Effects:** How substantial are the desirable anticipated effects? |  |  | - Trivial - Small - Moderate - Large - Varies - Don’t Know |
| **Undesirable Effects**: How substantial are the undesirable anticipated effects? |  |  | - Large - Moderate - Small - Trivial - Varies - Don’t Know |
| **Certainty of Evidence**: What is the overall certainty of the evidence of effects? |  |  | - Very Low - Low - Moderate - High - No Included Studies |
| **Value**: Is there important uncertainty about or variability in how much people value the main outcomes? |  |  | - Important Uncertainty or Variability - Possible Uncertainty or Variability - Probably No Important Uncertainty or Variability - No Important Variability or Uncertainty |
| **Balance of Effects**: Does the balance between desirable and undesirable effects favor the intervention or the comparison? |  |  | - Favors Comparison - Probably Favors the Comparison - Does Not Favor Either the Intervention or Comparison - Probably Favors the Intervention - Favors Intervention - Varies - Don’t Know |
| **Resources Required**: How large are the resource requirements (costs)? |  |  | - Large Costs - Moderate Costs - Negligible Costs or Savings - Moderate Savings - Large Savings - Varies - Don’t Know |
| **Certainty of Evidence of Required Resources**: What is the certainty of the evidence of resource requirements (costs)? |  |  | - Very Low - Low - Moderate - High - No Included Studies |
| **Cost-Effectiveness**: Does the cost-effectiveness of the intervention favor the intervention or the comparison? |  |  | - Favors Comparison - Probably Favors the Comparison - Does Not Favor Either the Intervention or Comparison - Probably Favors the Intervention - Favors Intervention - Varies - No Included Studies |
| **Equity**: What would be the impact on health equity? |  |  | - Reduced - Probably Reduced - Probably No Impact - Probably Increased - Increased - Varies - Don’t Know |
| **Acceptability**: Is the intervention acceptable to key stakeholders? |  |  | - No - Probably No - Probably Yes - Yes - Varies - Don’t Know |
| **Feasibility**: Is the intervention feasible to implement? |  |  | - No - Probably No - Probably Yes - Yes - Varies - Don’t Know |
| **Overall Recommendations**   - Strong Recommendation Against the Intervention - Conditional Recommendation Against the Intervention - Conditional Recommendation for Either the Intervention or the Comparison - Conditional Recommendation for the Intervention - Strong Recommendation for Intervention | | | |

| **Supplementary Table 3: List of Excluded Recommendations** | | |
| --- | --- | --- |
| **Congestive Heart Failure** | | |
| **S.No** | **Recommendation** | **Reason for Exclusion** |
| 1 | In patients hospitalized for HF, measurement of BNP or NT-pro BNP levels at admission is recommended to establish prognosis.  (COR 1; A) | In hospital recommendation |
| 2 | In patients hospitalized for HF, a predischarge BNP or NT-pro BNP level can be useful to inform the trajectory of the patient and establish a post discharge prognosis.  (COR 2A; B-NR) | In hospital recommendation |
| 3 | In patients with HF, endomyocardial biopsy may be useful when a specific diagnosis is suspected that would influence therapy.  (COR 2A; B-NR) | Unavailable in Pakistan |
| 4 | In selected adult patients with NYHA class III HF and history of a HF hospitalization in the past year or elevated natriuretic peptide levels, on maximally tolerated stable doses of GDMT with optimal device therapy, the use- fulness of wireless monitoring of PA pressure by an implanted hemodynamic monitor to reduce the risk of subsequent HF hospitalizations is uncertain.  (COR 2B; B-R) | Unavailable in Pakistan |
| 5 | In patients with NYHA class III HF with a HF hospitalization within the previous year, wireless monitoring of the PA pressure by an implanted hemodynamic monitor provides uncertain value.  (Value Statement: Uncertain Value B-NR) | Unavailable in Pakistan |
| 6 | In patients with HF who experience hyperkalemia (serum potassium level ≥5.5 mEq/L) while taking a renin-angiotensin-aldosterone system inhibitor (RAASi), the effectiveness of potassium binders (patiromer, sodium zirconium cyclosilicate) to improve outcomes by facilitating continuation of RAASi therapy is uncertain.  (COR 2B; B-R) | Unavailable in Pakistan |
| 7 | In selected high-risk patients with HFrEF and recent worsening of HF already on GDMT, an oral soluble guanylate cyclase stimulator (vericiguat) may be considered to reduce HF hospitalization and cardiovascular death,  (COR 2B; B-R) | Unavailable in Pakistan |
| 8 | In select patients with wild-type or variant trans- thyretin cardiac amyloidosis and NYHA class I to III HF symptoms, transthyretin tetramer sta- bilizer therapy (tafamidis) is indicated to reduce cardiovascular morbidity and mortality.  (COR 1; B-R) | Unavailable in Pakistan |
| 9 | At 2020 list prices, tafamidis provides low economic value (>$180 000 per QALY gained) in patients with HF with wild-type or variant transthyretin cardiac amyloidosis.  (Value Statement: Low Value B-NR) | Unavailable in Pakistan |
| 10 | In patients with advanced (stage D) HF refractory to GDMT and device therapy who are eligible for and awaiting MCS or cardiac trans- plantation, continuous intravenous inotropic support is reasonable as “bridge therapy.”  (COR 2A; B-NR) | In hospital recommendation |
| 11 | In select patients with stage D HF, despite optimal GDMT and device therapy who are ineligible for either MCS or cardiac transplantation, continuous intravenous inotropic sup- port may be considered as palliative therapy for symptom control and improvement in functional status.  (COR 2B; B-NR) | In hospital recommendation |
| 12 | In patients with HF, long-term use of either continuous or intermittent intravenous inotropic agents, for reasons other than palliative care or as a bridge to advanced therapies, is potentially harmful.  (COR 3:Harm; B-R) | In hospital recommendation |
| 13 | In select patients with advanced HFrEF with NYHA class IV symptoms who are deemed to be dependent on continuous intravenous inotropes or temporary MCS, durable LVAD implantation is effective to improve functional status, QOL, and survival.  (COR 1; A) | In hospital recommendation |
| 14 | In select patients with advanced HFrEF who have NYHA class IV symptoms despite GDMT, durable MCS can be beneficial to improve symptoms, improve functional class, and reduce mortality.  (COR 2A; B-R) | In hospital recommendation |
| 15 | In patients with advanced HFrEF who have NYHA class IV symptoms despite GDMT, durable MCS devices provide low to intermediate economic value based on current costs and outcomes.20–24 (Value Statement: Uncertain Value B-NR) | In hospital recommendation |
| 16 | In patients with advanced HFrEF and hemodynamic compromise and shock, temporary MCS, including percutaneous and extracorporeal ventricular assist devices, are reasonable as a “bridge to recovery” or “bridge to decision.”  (COR 2A; B-NR) | In hospital recommendation |
| 17 | For selected patients with advanced HF despite GDMT, cardiac transplantation is indicated to improve survival and QOL.  (COR 1; C-LD) | In hospital recommendation |
| 18 | In patients with stage D (advanced) HF despite GDMT, cardiac transplantation provides inter- mediate economic value.  (Value Statement: Intermediate Value C-LD) | In hospital recommendation |
| 19 | In patients hospitalized with HF, severity of con- gestion and adequacy of perfusion should be assessed to guide triage and initial therapy.  (COR 1; C-LD) | In hospital recommendation |
| 20 | In patients hospitalized with HF, the common precipitating factors and the overall patient trajectory should be assessed to guide appropriate therapy.  (COR 1; C-LD) | In hospital recommendation |
| 21 | For patients admitted with HF, treatment should address reversible factors, establish optimal volume status, and advance GDMT toward targets for outpatient therapy.  (COR 1; C-LD) | In hospital recommendation |
| 22 | In patients with HFrEF requiring hospitalization, preexisting GDMT should be continued and optimized to improve outcomes, unless contra- indicated.  (COR 1; B-NR) | In hospital recommendation |
| 23 | In patients experiencing mild decrease of renal function or asymptomatic reduction of blood pressure during HF hospitalization, diuresis and other GDMT should not routinely be discontinued.  (COR 1; B-NR) | In hospital recommendation |
| 24 | In patients with HFrEF, GDMT should be initiated during hospitalization after clinical stability is achieved.  (COR 1; B-NR) | In hospital recommendation |
| 25 | In patients with HFrEF, if discontinuation of GDMT is necessary during hospitalization, it should be reinitiated and further optimized as soon as possible.  (COR 1; B-NR) | In hospital recommendation |
| 26 | Patients with HF admitted with evidence of significant fluid overload should be promptly treated with intravenous loop diuretics to improve symptoms and reduce morbidity.  (COR 1; B-NR) | In hospital recommendation |
| 27 | For patients hospitalized with HF, therapy with diuretics and other guideline-directed medications should be titrated with a goal to resolve clinical evidence of congestion to reduce symptoms and rehospitalizations.  (COR 1; B-NR) | In hospital recommendation |
| 28 | For patients requiring diuretic treatment during hospitalization for HF, the discharge regimen should include a plan for adjustment of diuretics to decrease rehospitalizations.  (COR 1; B-NR) | In hospital recommendation |
| 29 | In patients hospitalized with HF when diuresis is inadequate to relieve symptoms and signs of congestion, it is reasonable to intensify the diuretic regimen using either: a. higher doses of intravenous loop diuretics.1,3); or b. addition of a second diuretic.  (COR 2A; B-NR) | In hospital recommendation |
| 30 | In patients who are admitted with decompensated HF, in the absence of systemic hypotension, intravenous nitro-glycerine or nitroprusside may be considered as an adjuvant to diuretic therapy for relief of dyspnoea.  (COR 2B; B-NR) | In hospital recommendation |
| 31 | In patients hospitalized with HF, prophylaxis for VTE is recommended to prevent venous thromboembolic disease.  (COR 1; B-R) | In hospital recommendation |
| 32 | In patients with cardiogenic shock, intravenous inotropic support should be used to maintain systemic perfusion and preserve end-organ performance.  (COR 1; B-NR) | In hospital recommendation |
| 33 | In patients with cardiogenic shock, temporary MCS is reasonable when end-organ function cannot be maintained by pharmacologic means to support cardiac function.  (COR 2A; B-NR) | In hospital recommendation |
| 34 | In patients with cardiogenic shock, management by a multidisciplinary team experienced in shock is reasonable.  (COR 2A; B-NR) | In hospital recommendation |
| 35 | In patients presenting with cardiogenic shock, placement of a PA line may be considered to define hemodynamic subsets and appropriate management strategies.  (COR 2B; B-NR) | In hospital recommendation |
| 36 | For patients who are not rapidly responding to initial shock measures, triage to centres that can provide temporary MCS may be considered to optimize management.  (COR 2B; C-LD) | In hospital recommendation |
| 37 | In patients hospitalized with worsening HF, participation in systems that allow benchmark- ing to performance measures is reasonable to increase use of evidence-based therapy, and to improve quality of care.  (COR 2A; B-NR) | In hospital recommendation |
| 38 | For patients with HF and symptoms caused by AF, AF ablation is reasonable to improve symptoms and QOL.  (COR 2A; B-R) | In hospital recommendation |
| 39 | For patients with AF and LVEF ≤50%, if a rhythm control strategy fails or is not desired, and ventricular rates remain rapid despite medical therapy, atrioventricular nodal ablation with implantation of a CRT device is reasonable.  (COR 2A; B-R) | In hospital recommendation |
| 40 | In patients with advanced HF with expected survival <6 months, timely referral to hospice can be useful to improve QOL.  (COR 2A; C-LD) | In hospital recommendation |
| **Stable Ischemic Heart Disease** | | |
| **S.No** | **Recommendation** | **Reason for Exclusion** |
| 1 | Patients with SIHD who have survived sudden cardiac death or potentially life-threatening ventricular arrhythmia should undergo coronary angiography to assess cardiac risk.  (Level of Evidence: B) | In hospital recommendation |
| 2 | Spinal cord stimulation may be considered for relief of refractory angina in patients with SIHD.  (Level of Evidence: C) | Unavailable in Pakistan |
| 3 | Transmyocardial revascularization (TMR) may be considered for relief of refractory angina in patients with SIHD.  (Level of Evidence: B) | Unavailable in Pakistan |
| 4 | Calculation of the STS and SYNTAX scores is reasonable in patients with unprotected left main and complex CAD.  (Level of Evidence: B) | In hospital recommendation |
| 5 | CABG to improve survival is recommended for patients with significant (50% diameter stenosis) left main coronary artery stenosis.  (Level of Evidence: B) | In hospital recommendation |
| 6 | PCI to improve survival is reasonable as an alternative to CABG in selected stable patients with significant (50% diameter stenosis) unprotected left main CAD with:  1) Anatomic conditions associated with a low risk of PCI procedural complications and a high likelihood of good long-term outcome (e.g., a low SYNTAX score [22], ostial or trunk left main CAD); and  2) Clinical characteristics that predict a significantly increased risk of adverse surgical outcomes (e.g., STS-predicted risk of operative mortality 5%)  (Level of Evidence: B) | In hospital recommendation |
| 7 | PCI to improve survival is reasonable in patients with UA/NSTEMI when an unprotected left main coronary artery is the culprit lesion, and the patient is not a candidate for CABG.  (Level of Evidence: B) | In hospital recommendation |
| 8 | PCI to improve survival is reasonable in patients with acute STEMI when an unprotected left main coronary artery is the culprit lesion, distal coronary flow is less than TIMI (Thrombolysis In Myocardial Infarction) grade 3, and PCI can be performed more rapidly and safely than CABG.  (Level of Evidence: C) | In hospital recommendation |
| 9 | PCI to improve survival may be reasonable as an alternative to CABG in selected stable patients with significant (50% diameter stenosis) unprotected left main CAD with:  a) anatomic conditions associated with a low to intermediate risk of PCI procedural complications and an intermediate to high likelihood of good long-term outcome (e.g., low–intermediate SYNTAX score of 33, bifurcation left main CAD); and  b) Clinical characteristics that predict an increased risk of adverse surgical outcomes (e.g., moderate–severe chronic obstructive pulmonary disease, disability from previous stroke, or previous cardiac surgery; STS-predicted risk of operative mortality 2%)  (Level of Evidence: B) | In hospital recommendation |
| 10 | PCI to improve survival should not be performed in stable patients with significant (50% diameter stenosis) unprotected left main CAD who have unfavourable anatomy for PCI and who are good candidates for CABG.  (Level of Evidence: B) | In hospital recommendation |
| 11 | CABG to improve survival is beneficial in patients with significant (70% diameter) stenoses in 3 major coronary arteries (with or without involvement of the proximal LAD artery) or in the proximal LAD artery plus 1 other major coronary artery.  (Level of Evidence: B) | In hospital recommendation |
| 12 | CABG or PCI to improve survival is beneficial in survivors of sudden cardiac death with presumed ischemia-mediated ventricular tachycardia caused by significant (70% diameter) stenosis in a major coronary artery.  (CABG Level of Evidence: B; PCI Level of Evidence: C) | In hospital recommendation |
| 13 | CABG to improve survival is reasonable in patients with significant (70% diameter) stenoses in 2 major coronary arteries with severe or extensive myocardial ischemia (e.g., high-risk criteria on stress testing, abnormal intracoronary hemodynamic evaluation, or 20% perfusion defect by myocardial perfusion stress imaging) or target vessels supplying a large area of viable myocardium.  (Level of Evidence: B) | In hospital recommendation |
| 14 | CABG to improve survival is reasonable in patients with mild– moderate LV systolic dysfunction (EF 35% to 50%) and significant (70% diameter stenosis) multivessel CAD or proximal LAD coronary artery stenosis, when viable myocardium is present in the region of intended revascularization.  (Level of Evidence: B) | In hospital recommendation |
| 15 | CABG with a left internal mammary artery (LIMA) graft to improve survival is reasonable in patients with significant (70% diameter) stenosis in the proximal LAD artery and evidence of extensive ischemia.  (Level of Evidence: B) | In hospital recommendation |
| 16 | It is reasonable to choose CABG over PCI to improve survival in patients with complex 3-vessel CAD (e.g., SYNTAX score 22), with or without involvement of the proximal LAD artery who are good candidates for CABG.  (Level of Evidence: B) | In hospital recommendation |
| 17 | CABG is probably recommended in preference to PCI to improve survival in patients with multivessel CAD and diabetes mellitus, particularly if a LIMA graft can be anastomosed to the LAD artery.  (Level of Evidence: B) | In hospital recommendation |
| 18 | The usefulness of CABG to improve survival is uncertain in patients with significant (70%) diameter stenoses in 2 major coronary arteries not involving the proximal LAD artery and without extensive ischemia.  (Level of Evidence: C) | In hospital recommendation |
| 19 | The usefulness of PCI to improve survival is uncertain in patients with 2- or 3-vessel CAD (with or without involvement of the proximal LAD artery) or 1-vessel proximal LAD disease.  (Level of Evidence: B) | In hospital recommendation |
| 20 | CABG might be considered with the primary or sole intent of improving survival in patients with SIHD with severe LV systolic dysfunction (EF 35%) whether or not viable myocardium is present.  (Level of Evidence: B) | In hospital recommendation |
| 21 | The usefulness of CABG or PCI to improve survival is uncertain in patients with previous CABG and extensive anterior wall ischemia on non-invasive testing.  (Level of Evidence: B) | In hospital recommendation |
| 22 | CABG or PCI should not be performed with the primary or sole intent to improve survival in patients with SIHD with 1 or more coronary stenoses that are not anatomically or functionally significant (e.g., 70% diameter non–left main coronary artery stenosis, FFR 0.80, no or only mild ischemia on non-invasive testing), involve only the left circumflex or right coronary artery, or subtend only a small area of viable myocardium.  (Level of Evidence: B) | In hospital recommendation |
| 23 | CABG or PCI to improve symptoms is beneficial in patients with 1 or more significant (70% diameter) coronary artery stenoses amenable to revascularization and unacceptable angina despite GDMT.  (Level of Evidence: A) | In hospital recommendation |
| 24 | CABG or PCI to improve symptoms is reasonable in patients with 1 or more significant (70% diameter) coronary artery stenoses and unacceptable angina for whom GDMT cannot be implemented because of medication contraindications, adverse effects, or patient preferences.  (Level of Evidence: C) | In hospital recommendation |
| 25 | PCI to improve symptoms is reasonable in patients with previous CABG, 1 or more significant (70% diameter) coronary artery stenoses associated with ischemia, and unacceptable angina despite GDMT.  (Level of Evidence: C) | In hospital recommendation |
| 26 | It is reasonable to choose CABG over PCI to improve symptoms in patients with complex 3-vessel CAD (e.g., SYNTAX score 22), with or without involvement of the proximal LAD artery, who are good candidates for CABG.  (Level of Evidence: B) | In hospital recommendation |
| 27 | CABG to improve symptoms might be reasonable for patients with previous CABG, 1 or more significant (70% diameter) coronary artery stenoses not amenable to PCI, and unacceptable angina despite GDMT.  (Level of Evidence: C) | In hospital recommendation |
| 28 | TMR performed as an adjunct to CABG to improve symptoms may be reasonable in patients with viable ischemic myocardium that is perfused by arteries that are not amenable to grafting.  (Level of Evidence: B) | In hospital recommendation |
| 29 | CABG or PCI to improve symptoms should not be performed in patients who do not meet anatomic (50% diameter left main or 70% non–left main stenosis diameter) or physiological (e.g., abnormal FFR) criteria for revascularization.  (Level of Evidence: C) | In hospital recommendation |
| 30 | PCI with coronary stenting (BMS or DES) should not be performed if the patient is not likely to be able to tolerate and comply with DAPT for the appropriate duration of treatment based on the type of stent implanted.  (Level of Evidence: B) | In hospital recommendation |
| 31 | Hybrid coronary revascularization (defined as the planned combination of LIMA-to-LAD artery grafting and PCI of 1 non-LAD coronary arteries) is reasonable in patients with 1 or more of the following (Level of Evidence: B):  a. Limitations to traditional CABG, such as heavily calcified proximal aorta or poor target vessels for CABG (but amenable to PCI)  b. Lack of suitable graft conduits  c. Unfavourable LAD artery for PCI (i.e., excessive vessel tortuosity or chronic total occlusion). | In hospital recommendation |
| 32 | Hybrid coronary revascularization (defined as the planned combination of LIMA-to-LAD artery grafting and PCI of 1 non-LAD coronary arteries) may be reasonable as an alternative to multivessel PCI or CABG in an attempt to improve the overall risk–benefit ratio of the procedures.  (Level of Evidence: C) | In hospital recommendation |

**Congestive Heart Failure**

**Adoloped from: 2022 AHA/ACC/HFSA Guideline for the Management of Heart Failure: A Report of the American College of Cardiology/American Heart Association Joint Committee on Clinical Practice Guidelines** **May 3, 2022, DOI: 10.1161/CIR.0000000000001063**

**Key to the understanding of Class of Recommendations and Levels of Evidence.**

| Classes of recommendation (COR) | | | | | | |
| --- | --- | --- | --- | --- | --- | --- |
| Class I | Class IIa | | Class IIb | Class III | | Class III |
| Strong Benefit >>> Risk | Moderate Benefit >> Risk | | Weak Benefit ≥ Risk | No benefit (Moderate) Benefit = Risk | | Harm (Strong) Risk > Benefit |
| Levels of evidence (LOE) | | | | | | |
| Level A | | Level B | | | Level C | |
| Multiple population evaluated | | Limited population evaluated | | | Very limited population evaluated | |

**Table of Recommendation**

|  | **Diagnosis** |
| --- | --- |
|  | **Initial serial evaluation** |
|  | **Clinical Assessment: History & Physical Examination** |
|  | In patients with HF, vital signs and evidence of clinical congestion should be assessed at each encounter to guide overall management, including adjustment of diuretics and other medications.  [COR 1; B-NR] |
|  | In patients with symptomatic HF, clinical factors indicating the presence of advanced HF should be sought via history and physical examination.  [COR 1; B-NR] |
|  | In patients with cardiomyopathy, a 3-generation family history should be obtained or updated when assessing the cause of the cardiomyopathy- to identify possible inherited disease.  [COR 1; B-NR] |
|  | In patients presenting with HF, a thorough history and physical examination should direct diagnostic strategies to uncover specific causes that may warrant disease-specific management.  [COR 1; B-NR] |
|  | In patients presenting with HF, a thorough history and physical examination should be obtained and performed to identify cardiac and noncardiac disorders, lifestyle and behavioural factors, and social determinants of health that might cause or accelerate the development or progression of HF. [COR 1; C-EO] |
|  | **Initial Laboratory and Electrocardiographic Testing** |
|  | For patients presenting with HF, the specific cause of HF should be explored using additional laboratory testing for appropriate management.  [COR 1; B-NR] |
|  | For patients who are diagnosed with HF, laboratory evaluation should include complete blood count, urinalysis, serum electrolytes, blood urea nitrogen, serum creatinine, glucose, lipid profile, liver function tests, iron studies, and thyroid-stimulating hormone to optimize management. [COR 1; C-EO] |
|  | For ALL patients presenting with HF, a 12-lead ECG should be performed at the initial encounter to optimize management.  [COR 1; C-EO] |
|  | **Use of Biomarkers for Prevention, Initial Diagnosis, and Risk Stratification** |
|  | In patients presenting with dyspnea, measurement of B-type natriuretic peptide (BNP) or N-terminal prohormone of B-type natriuretic peptide (NT-proBNP) is useful to support a diagnosis or exclusion of HF.  [COR 1; A] |
|  | In patients with chronic HF, measurements of BNP or NT-proBNP levels are recommended for risk stratification.  [COR 1; A] |
|  | In patients at risk of developing HF, BNP or NT-pro BNP based screening followed by team-based care, including a cardiovascular specialist, can be useful to prevent the development of LV dysfunction or new-onset HF.  [COR 2A; B-R] |
|  | **Evaluation With Cardiac Imaging** |
|  | In patients with suspected or new-onset HF, or those presenting with acute decompensated HF, a chest x-ray should be performed to assess heart size and pulmonary congestion and to detect alternative cardiac, pulmonary, and other diseases that may cause or contribute to the patient’s symptoms.  [COR 1; C-LD] |
|  | In patients with suspected or newly diagnosed, transthoracic echocardiography (TTE) should be performed during initial evaluation to assess cardiac structure and function.  [COR 1; C-LD] |
|  | In patients with HF who have had a significant clinical change, or who have received GDMT and are being considered for invasive procedures or device therapy, repeat measurement of EF, degree of structural remodeling, and valvular function are useful to inform therapeutic interventions.  [COR 1; C-LD] |
|  | In patients for whom echocardiography is inadequate, alternative imaging (eg, cardiac magnetic resonance [CMR], cardiac computed tomography [CT], radionuclide imaging) is recommended assessment of LVEF.  [COR 1; C-LD] |
|  | In patients with HF or cardiomyopathy, CMR can be useful for diagnosis or management.  [COR 2A; B-NR] |
|  | In patients with HF, an evaluation for possible ischemic heart disease can be useful to identify the cause and guide management.  [COR 2A; B-NR] |
|  | In patients with HF and coronary artery disease (CAD) who are candidates for coronary revascularization, noninvasive stress imaging (stress echocardiography, single-photon emission CT [SPECT], CMR, or positron emission totomography PET]) may be considered for detection of myocardial ischemia to help guide coronary revascularization.  [COR 2B; B-NR] |
|  | In patients with HF in the absence of 1) clinical status change, 2) treatment interventions that might have had a signiﬁcant eﬀect on cardiac function, or 3) candidacy for invasive procedures or device therapy, routine repeat assessment of LV function is NOT indicated.  [COR 3: No Benefit; C-EO] |
|  | **Invasive Evaluation** |
|  | In selected patients with HF with persistent or worsening symptoms, signs, diagnostic parameters, and in whom hemodynamics are uncertain invasive hemodynamic monitoring can be useful to guide management.  [COR 2A; C-EO] |
|  | In patients with HF, routine use of invasive hemodynamic monitoring is NOT recommended.  [COR 3: No Benefit; B-R] |
|  | For patients undergoing routine evaluation of HF, endomyocardial biopsy should NOT be performed because of the risk of complications.  [COR 3: Harm; C-LD] |
|  | **Exercise and Functional Capacity Testing** |
|  | In patients with HF, assessment and documentation of NYHA functional classification are recommended to determine eligibility for treatments.  [COR 1; C-LD] |
|  | In selected ambulatory patients with HF, cardiopulmonary exercise testing (CPET) is recommended to determine appropriateness of advanced treatments (e.g., LVAD, heart transplant).  [COR 1; C-LD] |
|  | In ambulatory patients with HF, performing a CPET or 6-minute walk test is reasonable to assess functional capacity.  [COR 2A; C-LD] |
|  | In ambulatory patients with unexplained dyspnea, CPET is reasonable to evaluate the cause of dyspnea.  [COR 2A; C-LD] |
|  | **Initial and Serial Evaluation: Clinical Assessment: HF Risk Scoring** |
|  | In ambulatory or hospitalized patients with HF, validated multivariable risk scores can be useful to estimate subsequent risk of mortality.  [COR 2A; B-NR] |
|  | **Cardiac Amyloidosis** |
|  | In patients with high clinical suspicion for cardiac amyloidosis, without evidence of serum or urine monoclonal light chains, bone scintigraphy should be performed to confirm the presence of transthyretin cardiac amyloidosis.  [COR 1; B-NR] |
|  | In patients for whom a diagnosis of transthyretin cardiac amyloidosis is made, genetic testing with TTR gene sequencing is recommended to differentiate hereditary variant from wild-type transthyretin cardiac amyloidosis.  [COR 1; B-NR] |
|  | **TREATMENT AND MANAGEMENT** |
|  | **Pharmacological Treatment for HFrEF** |
|  | **Renin-Angiotensin System Inhibition with ACEi or ARB or ARNi** |
|  | In patients with HFrEF and NYHA class II to III symptoms, the use of ARNi is recommended to reduce morbidity and mortality.  [COR 1; A] |
|  | In patients with previous or current symptoms of chronic HFrEF, the use of ACEi is beneﬁcial to reduce morbidity and mortality when the use of ARNi is not feasible.  [COR 1; A] |
|  | In patients with previous or current symptoms of chronic HFrEF who are intolerant to ACEi because of cough or angioedema and when the use of ARNi is not feasible, the use of ARB is recommended to reduce morbidity and mortality.  [COR 1; A] |
|  | In patients with previous or current symptoms of chronic HFrEF, in whom ARNi is not feasible, treatment with an ACEi or ARB provides high economic value.  [Value Statement: High Value A] |
|  | In patients with chronic symptomatic HFrEF NYHA class II or III who tolerate an ACEi or ARB, replacement by an ARNi is recommended to further reduce morbidity and mortality.  [COR 1; B-R] |
|  | In patients with chronic symptomatic HFrEF, treatment with an ARNi instead of an ACEi provides high economic value.  [Value Statement: High Value A] |
|  | ARNi should NOT be administered concomitantly with ACEi or within 36 hours of the last dose of an ACEi.  [COR 3: Harm; B-R] |
|  | ARNi should NOT be administered to patients with any history of angioedema.  [COR 3: Harm; C-LD] |
|  | ACEi should NOT be administered to patients with any history of angioedema.  [COR 3: Harm; C-LD] |
|  | **Beta Blockers** |
|  | In patients with HFrEF, with current or previous symptoms, use of 1 of the 3 beta blockers proven to reduce mortality (e.g., bisoprolol, carvedilol, sustained-release metoprolol succinate) is recommended to reduce mortality and hospitalizations.  [COR 1; A] |
|  | In patients with HFrEF, with current or previous symptoms, beta-blocker therapy provides high economic value.  [Value Statement: High Value A] |
|  | **Mineralocorticoid Receptor Antagonists (MRAs)** |
|  | In patients with HFrEF and NYHA class II to IV symptoms, an MRA (spironolactone or eplere- none) is recommended to reduce morbidity and mortality, if eGFR is >30 mL/min/1.73 m2 and serum potassium is <5.0 mEq/L. Careful monitoring of potassium, renal function, and diuretic dosing should be performed at initiation and closely monitored thereafter to minimize risk of hyperkalemia and renal insufficiency.  [COR 1; A] |
|  | In patients with HFrEF and NYHA class II to IV symptoms, MRA therapy provides high economic value.  (Value Statement: High Value A) |
|  | In patients taking MRA whose serum potassium cannot be maintained at <5.5 mEq/L, MRA should be DISCONTINUED to avoid life-threatening hyperkalemia.  [COR 3: Harm; B-NR] |
|  | **Sodium-Glucose Cotransporter 2 Inhibitors** |
|  | In patients with symptomatic chronic HFrEF, SGLT2i are recommended to reduce hospitalization for HF and cardiovascular mortality, irrespective of the presence of type 2 diabetes.  [COR 1; A] |
|  | In patients with symptomatic chronic HFrEF, SGLT2i therapy provides intermediate economic value.  [Value Statement: High Value A] |
|  | **Hydralazine and Isosorbide Dinitrate** |
|  | In patients with current or previous symptomatic HFrEF who cannot be given first-line agents, such as ARNi, ACEi, or ARB, because of drug intolerance or renal insuﬃciency, a combination of hydralazine and isosorbide dinitrate might be considered to reduce morbidity and mortality.  [COR 2B; C-LD] |
|  | **Other Drug Treatment** |
|  | In patients with HF class II to IV symptoms, omega-3 polyunsaturated fatty acid (PUFA) supplementation may be reasonable to use as adjunctive therapy to reduce mortality and cardio- vascular hospitalizations.  [COR 2B; B-R] |
|  | In patients with chronic HFrEF without a specific indication (e.g., venous thromboembolism [VTE], AF, a previous thromboembolic event, or a cardioembolic source), anticoagulation is NOT recommended.  [COR 3: No Benefit; B-R] |
|  | **Drugs of Unproven Value or That May Worsen HF** |
|  | In patients with HFrEF, dihydropyridine calcium channel-blocking drugs are NOT recommended treatment for HF.  (COR 3: No Benefit; A) |
|  | In patients with HFrEF, vitamins, nutritional supplements, and hormonal therapy are NOT recommended other than to correct specific deficiencies.  [COR 3: No Benefit; B-R] |
|  | In patients with HFrEF, nondihydropyridine calcium channel-blocking drugs are NOT recommended.  [COR 3: Harm; A] |
|  | In patients with HFrEF, class IC antiarrhythmic medications and dronedarone may increase the risk of mortality.  [COR 3: Harm; A] |
|  | In patients with HFrEF, thiazolidinediones increase the risk of worsening HF symptoms and hospitalizations.  [COR 3: Harm; A] |
|  | In patients with type 2 diabetes and high cardiovascular risk, the dipeptidyl peptidase-4 (DPP-4) inhibitors saxagliptin and alogliptin increase the risk of HF hospitalization and should be AVOIDED in patients with HF.  [COR 3: Harm; B-R] |
|  | In patients with HFrEF, NSAIDs worsen HF symptoms and should be AVOIDED or withdrawn whenever possible.  [COR 3: Harm; B-NR] |
|  | **GDMT Dosing: Sequencing and Uptitration** |
|  | In patients with HFrEF, titration of guideline-directed medication dosing to achieve target doses showed to be eﬃcacious in RCTs is recommended, to reduce cardiovascular mortality and HF hospitalizations, unless not well tolerated.  [COR 1; A] |
|  | In patients with HFrEF, titration and optimization of guideline-directed medications as frequently as every 1 to 2 weeks depending on the patient’s symptoms, vital signs, and laboratory findings can be useful to optimize management.  [COR 2A; C-EO] |
|  | **Additional Medical Therapies** |
|  | For patients with symptomatic (NYHA class II to III) stable chronic HFrEF (LVEF ≤35%) who are receiving GDMT, including a beta blocker at the maximum tolerated dose, and who are in sinus rhythm with a heart rate of ≥70 bpm at rest, ivabradine can be beneﬁcial to reduce HF hospitalizations and cardiovascular death.  [COR 2A; B-R] |
|  | **Pharmacological Treatment for Stage CHFrEF: Digoxin** |
|  | In patients with symptomatic HFrEF despite GDMT (or who are unable to tolerate GDMT), digoxin might be considered to decrease hospitalizations for HF.  [COR 2B; B-R] |
|  | **ICDs and CRTs** |
|  | In patients with nonischaemic DCM or ischemic heart disease at least 40 days post-MI with LVEF ≤ 35% and NYHA class II or III symptoms on chronic GDMT, who have a reasonable expectation of meaningful survival for >1 year, ICD therapy is recommended for primary prevention of SCD to reduce total mortality.  [COR 1; A] |
|  | A transvenous ICD provides high economic value in the primary prevention of SCD particularly when the patient’s risk of death caused by ventricular arrhythmia is deemed high and the risk of non-arrhythmic death (either cardiac or noncardiac) is deemed low based on the patient’s burden of comorbidities and functional status.  [Value Statement: High Value A] |
|  | In patients at least 40 days post-MI with LVEF ≤30% and NYHA class I symptoms while receiving GDMT, who have a reasonable expectation of meaningful survival for >1 year, ICD therapy is recommended for primary prevention of SCD to reduce total mortality.  [COR 1; B-R] |
|  | For patients who have LVEF ≤35%, sinus rhythm, left bundle branch block (LBBB) with a QRS duration ≥150 ms, and NYHA class II, III, or ambulatory IV symptoms on GDMT, CRT is indicated to reduce total mortality, reduce hospitalizations, and improve symptoms and QOL.  [COR 1; B-R] |
|  | For patients who have LVEF ≤35%, sinus rhythm, LBBB with a QRS duration of ≥150 ms, and NYHA class II, III, or ambulatory IV symptoms on GDMT, CRT implantation provides high economic value.  [Value Statement: High Value A] |
|  | For patients who have LVEF ≤35%, sinus rhythm, a non-LBBB pattern with a QRS duration ≥150 ms, and NYHA class II, III, or ambulatory class IV symptoms on GDMT, CRT can be useful to reduce total mortality, reduce hospitalizations, and improve symptoms and QOL.  [COR 2A; B-R] |
|  | In patients with high-degree or complete heart block and LVEF of 36% to 50%, CRT is reasonable to reduce total mortality, reduce hospitalizations, and improve symptoms and QOL.  [COR 2A; B-R] |
|  | For patients who have LVEF ≤35%, sinus rhythm, LBBB with a QRS duration of 120 to 149 ms, and NYHA class II, III, or ambulatory IV symptoms on GDMT, CRT can be useful to reduce total mortality, reduce hospitalizations, and improve symptoms and QoL.  [COR 2A; B-NR] |
|  | In patients with AF and LVEF ≤35% on GDMT, CRT can be useful to reduce total mortality, improve symptoms and QOL, and increase LVEF, if: a) the patient requires ventricular pacing or otherwise meets CRT criteria and b) atrioventricular nodal ablation or pharmacological rate control will allow near 100% ventricular pacing with CRT.  [COR 2A; B-NR] |
|  | For patients on GDMT who have LVEF ≤35% and are undergoing placement of a new or replacement device implantation with anticipated requirement for significant (>40%) ventricular pacing, CRT can be useful to reduce total mortality, reduce hospitalizations, and improve symptoms and QOL.  [COR 2A; B-NR] |
|  | In patients with genetic arrhythmogenic cardiomyopathy with high-risk features of sudden death, with EF ≤45%, implantation of ICD is reasonable to decrease sudden death.  [COR 2A; B-NR] |
|  | For patients who have LVEF ≤35%, sinus rhythm, a non-LBBB pattern with QRS duration of 120 to 149 ms, and NYHA class III or ambulatory class IV on GDMT, CRT may be considered to reduce total mortality, reduce hospitalizations, and improve symptoms and QOL.  [COR 2B; B-NR] |
|  | For patients who have LVEF ≤30%, ischemic cause of HF, sinus rhythm, LBBB with a QRS duration ≥150 ms, and NYHA class I symptoms on GDMT, CRT may be considered to reduce hospitalizations and improve symptoms and QOL.  [COR 2B; B-NR] |
|  | In patients with QRS duration <120 ms, CRT is NOT recommended.  [COR 3: No Benefit; B-R] |
|  | For patients with NYHA class I or II symptoms and non-LBBB patterns with QRS duration  <150 ms, CRT is NOT recommended.  [COR 3: No Benefit; B-NR] |
|  | For patients whose comorbidities or frailty limit survival with the good functional capacity to  <1 year, ICD and cardiac resynchronization therapy with defibrillation (CRT-D) are NOT indicated.  [COR 3:No Benefit; C-LD] |
|  | **Revascularization for CAD** |
|  | In selected patients with HF, reduced EF (EF ≤35%), and suitable coronary anatomy, surgical revascularization plus GDMT is beneficial to improve symptoms, cardiovascular hospitalizations, and long-term all-cause mortality.  [COR 1; B-R] |
|  | **Valvular Heart Disease** |
|  | In patients with HF, VHD should be managed in a multidisciplinary manner in accordance with clinical practice guidelines for VHD to prevent worsening of HF and adverse clinical outcomes.  [COR 1; B-R] |
|  | In patients with chronic severe secondary MR and HFrEF, optimization of GDMT is recommended before any intervention for secondary MR related to LV dysfunction.  [COR 1; C-LD] |
|  | **HF With Mildly Reduced Ejection Fraction** |
|  | In patients with HFmrEF, SGLT2i can be beneficial in decreasing HF hospitalizations and cardio- vascular mortality.  [COR 2A; B-R] |
|  | Among patients with current or previous symptomatic HFmrEF (LVEF, 41%–49%), use of evidence-based beta-blockers for HFrEF, ARNi, ACEi, or ARB, and MRAs may be considered to reduce the risk of HF hospitalization and cardiovascular mortality, particularly among patients with LVEF on the lower end of this spectrum.  [COR 2B; B-NR] |
|  | **HF With Improved Ejection Fraction** |
|  | In patients with HFimpEF after treatment, GDMT should be continued to prevent relapse of HF and LV dysfunction, even in patients who may become asymptomatic.  [COR 1; B-R] |
|  | **Preserved EF (HFpEF)** |
|  | Patients with HFpEF and hypertension should have medication titrated to attain blood pressure targets in accordance with published clinical practice guidelines to prevent morbidity.  [COR 1; C-LD] |
|  | In patients with HFpEF, SGLT2i can be beneficial in decreasing HF hospitalizations and cardiovascular mortality.  [COR 2A; B-R] |
|  | In patients with HFpEF, management of AF can be useful to improve symptoms.  [COR 2A; C-EO] |
|  | In selected patients with HFpEF, MRAs may be considered to decrease hospitalizations, particularly among patients with LVEF on the lower end of this spectrum.  [COR 2B; B-R] |
|  | In selected patients with HFpEF, the use of ARB may be considered to decrease hospitalizations, particularly among patients with LVEF on the lower end of this spectrum.  [COR 2B; B-R] |
|  | In selected patients with HFpEF, ARNi may be considered to decrease hospitalizations, particularly among patients with LVEF on the lower end of this spectrum.  [COR 2B; B-R] |
|  | In patients with HFpEF, routine use of nitrates or phosphodiesterase-5 inhibitors to increase activity or QOL is ineffective.  [COR 3: No Benefit; B-R] |
|  | **Management of Stage B: Preventing the Syndrome of Clinical HF in Patients With Pre-HF** |
|  | In patients with LVEF ≤40%, ACEi should be used to prevent symptomatic HF and reduce mortality.  [COR 1; A] |
|  | In patients with a recent or remote history of MI or ACS, statins should be used to prevent symptomatic HF and adverse cardiovascular events.  [COR 1; A] |
|  | In patients with a recent MI and LVEF ≤40% who are intolerant to ACEi, ARB should be used to prevent symptomatic HF and reduce mortality.  [COR 1; B-R] |
|  | In patients with a recent or remote history of MI or acute coronary syndrome (ACS) and LVEF ≤  40%, evidence-based beta blockers should be used to reduce mortality.  [COR 1; B-R] |
|  | In patients who are at least 40 days post-MI with LVEF ≤30% and NYHA class I symptoms while receiving GDMT and have reasonable expectation of meaningful survival for >1 year, an ICD is recommended for primary prevention of sudden cardiac death (SCD) to reduce total mortality.  [COR 1; B-R] |
|  | In patients with LVEF ≤40%, beta blockers should be used to prevent symptomatic HF.  [COR 1; C-LD] |
|  | In patients with LVEF <50%, thiazolidinediones should NOT be used because they increase the risk of HF, including hospitalizations.  [COR 3: Harm; B-R] |
|  | In patients with LVEF <50%, non-dihydropyridine calcium channel blockers with negative inotropic effects may be harmful.  [COR 3: Harm; C-LD] |
|  | **Treatment of Cardiac Amyloidosis** |
|  | In patients with cardiac amyloidosis and AF, anticoagulation is reasonable to reduce the risk of stroke regardless of the CHA2DS2-VASc (congestive heart failure, hypertension, age ≥75 years, diabetes mellitus, stroke or transient ischemic attack [TIA], vascular disease, age 65 to 74 years, sex category) score.  [COR 2A; C-LD] |
|  | **Nonpharmacological Management: Advanced HF** |
|  | For patients with advanced HF and hyponatremia, the benefit of fluid restriction to reduce congestive symptoms is uncertain.  [COR 2B; C-LD] |
|  | **Management of anemia or iron deficiency** |
|  | In patients with HFrEF and iron deﬁciency with or without anemia, intravenous iron replacement  is reasonable to improve functional status and QOL.  [COR 2A; B-R] |
|  | In patients with HF and anemia, erythropoietin-stimulating agents should NOT be used to improve morbidity and mortality.  [COR 3:Harm; B-R] |
|  | **Management of hypertension** |
|  | In patients with HFrEF and hypertension, up-titration of GDMT to the maximally tolerated target dose is recommended.  [COR 1; C-LD] |
|  | **Management of sleep disorders** |
|  | In patients with HF and suspicion of sleep-disordered breathing, a formal sleep assessment is reasonable to confirm the diagnosis and differentiate between obstructive and central sleep apnea.  [COR 2A; C-LD] |
|  | In patients with HF and obstructive sleep apnea, continuous positive airway pressure may be reasonable to improve sleep quality and decrease daytime sleepiness.  [COR 2A; B-R] |
|  | In patients with NYHA class II to IV HFrEF and central sleep apnea, adaptive servo-ventilation causes harm.  [COR 3:Harm; B-R] |
|  | **Management of diabetes** |
|  | In patients with HF and type 2 diabetes, the use of SGLT2i is recommended for the management of hyperglycaemia and to reduce HF-related morbidity and mortality.  [COR 1; A] |
|  | **Management of AF in HF** |
|  | Patients with chronic HF with permanent-persistent-paroxysmal AF and a CHA2DS2-VASc score of ≥2 (for men) and ≥3 (for women) should receive chronic anticoagulant therapy.  [COR 1; A] |
|  | For patients with chronic HF with permanent- persistent-paroxysmal AF, DOAC is recommended over warfarin in eligible patients.  [COR 1; A] |
|  | For patients with chronic HF and permanent- persistent-paroxysmal AF, chronic anticoagulant therapy is reasonable for men and women without additional risk factors.  [COR 2A; B-NR] |
|  | **Cardio-Oncology** |
|  | In patients who develop cancer therapy-related cardiomyopathy or HF, a multidisciplinary discussion involving the patient about the risk-benefit ratio of cancer therapy interruption, discontinuation, or continuation is recommended to improve management.  [COR 1; B-NR] |
|  | In asymptomatic patients with cancer therapy-related cardiomyopathy (EF <50%), ARB, ACEi, and beta blockers are reasonable to prevent progression to HF and improve cardiac function.  [COR 2A; B-NR] |
|  | In patients with cardiovascular risk factors or known cardiac disease being considered for potentially cardiotoxic anticancer therapies, pretherapy evaluation of cardiac function is reasonable to establish baseline cardiac function and guide the choice of cancer therapy.  [COR 2A; B-NR] |
|  | **Management of AF in HF** |
|  | In patients with cardiovascular risk factors or known cardiac disease receiving potentially cardio- toxic anticancer therapies, monitoring of cardiac function is reasonable for the early identification of drug-induced cardiomyopathy.  [COR 2A; B-NR] |
|  | In patients at risk of cancer therapy-related cardiomyopathy, initiation of beta blockers and ACEi/ARB for the primary prevention of drug-induced cardiomyopathy is of uncertain benefit.  [COR 2B; B-R] |
|  | In patients being considered for potentially cardiotoxic therapies, serial measurement of cardiac troponin might be reasonable for further risk stratification.  [COR 2B; C-LD] |
|  | **HF and Pregnancy** |
|  | In women with a history of HF or cardiomyopathy, including previous peripartum cardiomyopathy, patient-centred counselling regarding contraception and the risks of cardiovascular deterioration during pregnancy should be provided.  [COR 1; C-LD] |
|  | In women with acute HF caused by peripartum cardiomyopathy and LVEF <30%, anticoagulation may be reasonable at diagnosis, until 6 to 8 weeks postpartum, although the efficacy and safety are uncertain.  [COR 2B; C-LD] |
|  | In women with HF or cardiomyopathy who are pregnant or currently planning for pregnancy, ACEi, ARB, ARNi, MRA, SGLT2i, ivabradine, and vericiguat should NOT be administered because of significant risks of foetal harm.  [COR 3:Harm ; C-LD] |
|  | **PROGNOSIS, OUTCOMES AND REFERRAL TO SPECIALIST** |
|  | **Integration of Care: Transitions and Team-Based Approaches** |
|  | In patients with high-risk HF, particularly those with recurrent hospitalizations for HFrEF, referral to multidisciplinary HF disease management programs is recommended to reduce the risk of hospitalization.  [COR 1; B-R] |
|  | In patients hospitalized with worsening HF, patient-centred discharge instructions with a clear  plan for transitional care should be provided before hospital discharge.  [COR 1; B-NR] |
|  | In patients being discharged after hospitalization for worsening HF, an early follow-up, generally within 7 days of hospital discharge, is reasonable to optimize care and reduce re-hospitalization.  [COR 2A; B-NR] |
|  | **Specialty Referral for Advanced HF** |
|  | In patients with advanced HF, when consistent with the patient’s goals of care, timely referral for HF specialty care is recommended to review HF management and assess suitability for advanced HF therapies (e.g., LVAD, cardiac transplantation, palliative care, and palliative inotropes).  [COR 1; C-LD] |
|  | **PREVENTION AND SCREENING** |
|  | **Patients at Risk for HF (Stage A: Primary Prevention)** |
|  | In patients with hypertension, blood pressure should be controlled in accordance with GDMT for hypertension to prevent symptomatic HF.  [COR 1; A] |
|  | In patients with type 2 diabetes and either established CVD or at high cardiovascular risk, SGLT2i should be used to prevent hospitalizations for HF.  [COR 1; A] |
|  | In the general population, healthy lifestyle habits such as regular physical activity, maintaining normal weight, healthy dietary patterns, and avoiding smoking are helpful to reduce future risk of HF.  [COR 1; B-NR] |
|  | For patients at risk of developing HF, natriuretic peptide biomarker–based screening followed by team-based care, including a cardiovascular specialist optimizing GDMT, can be useful to prevent the development of LV dysfunction (systolic or diastolic) or new-onset HF.  [COR 2A; B-R] |
|  | In the general population, validated multivariable risk scores can be useful to estimate subsequent risk of incident HF.  [COR 2A; B-NR] |
|  | **Cardiac Amyloidosis** |
|  | Patients for whom there is a clinical suspicion for cardiac amyloidosis, should have screening for serum and urine monoclonal light chains with serum and urine immuno- fixation electrophoresis and serum free light chains.  [COR 1; B-NR] |
|  | **Patient Education** |
|  | **Nonpharmacological Interventions** |
|  | Patients with HF should receive care from multidisciplinary teams to facilitate the implementation of GDMT, address potential barriers to self-care, reduce the risk of subsequent rehospitalization for HF, and improve survival.  [COR 1; A] |
|  | Patients with HF should receive specific education and support to facilitate HF self-care in a multidisciplinary manner.  [COR 1; B-R] |
|  | In patients with HF, vaccinating against respiratory illnesses is reasonable to reduce mortality.  [COR 2A; B-NR] |
|  | In adults with HF, screening for depression, social isolation, frailty, and low health literacy as risk factors for poor self-care is reasonable to improve management.  [COR 2A; B-NR] |
|  | **Dietary Sodium Restriction** |
|  | For patients with stage C HF, avoiding excessive sodium intake is reasonable to reduce congestive symptoms.  [COR 2A; C-LD] |
|  | **Management of Stage C HF: Activity, Exercise Prescription, and Cardiac Rehabilitation** |
|  | For patients with HF who can participate, exercise training (or regular physical activity) is recommended to improve functional status, exercise performance, and QOL.  [COR 1; A] |
|  | In patients with HF, a cardiac rehabilitation program can be useful to improve functional capacity, exercise tolerance, and health related QOL.  [COR 2A; B-NR] |
|  | **Diuretics and Decongestion Strategies in Patients With HF** |
|  | In patients with HF who have fluid retention, diuretics are recommended to relieve congestion, improve symptoms, and prevent worsening HF.  [COR 1; B-NR] |
|  | For patients with HF and congestive symptoms, addition of a thiazide (eg, metolazone) to a loop diuretic should be reserved for patients who do not respond to moderate- or high-dose loop diuretics to minimize electrolyte abnormalities.  [COR 1; B-NR] |
|  | **Genetic Evaluation and Testing** |
|  | In first-degree relatives of selected patients with genetic or inherited cardiomyopathies, genetic screening and counselling are recommended to detect cardiac disease and prompt consideration of treatments to decrease HF progression and sudden death.  [COR 1; B-NR] |
|  | In select patients with nonischaemic cardiomyopathy, referral for genetic counselling and testing is reasonable to identify conditions that could guide treatment for patients and family members.  [COR 2A; B-NR] |
|  | **FOLLOW UP** |
|  | For all patients with HF, palliative and supportive care including high-quality communication, conveyance of prognosis, clarifying goals of care, shared decision-making, symptom management, and caregiver support should be provided to improve QOL and relieve suffering.  [COR 1; C-LD] |
|  | For patients with HF being considered for, or treated with life-extending therapies, the option for discontinuation should be anticipated and discussed through the continuum of care, including at the time of initiation, and reassessed with changing medical conditions and shifting goals of care.  [COR 1; C-LD] |
|  | For patients with HF particularly stage D  HF patients being evaluated for advanced therapies, patients requiring inotropic support or temporary mechanical support, patients experiencing uncontrolled symptoms, major medical decisions, or multimorbidity, frailty, and cognitive impairment—specialist palliative care consultation can be useful to improve QOL and relieve suffering.  [COR 2A; B-R] |
|  | For patients with HF, execution of advance care directives can be useful to improve documentation of treatment preferences, delivery of patient-centred care, and dying in preferred place.  [COR 2A; C-LD] |
|  | In patients with HF, standardized assessment of patient-reported health status using a validated questionnaire can be useful to provide incremental information for patient functional status, symptom burden, and prognosis.  [COR 2A; C-LD] |
|  | **Disparities and Vulnerable Populations** |
|  | In vulnerable patient populations at risk for health disparities, HF risk assessments and multidisciplinary management strategies should target both known risks for CVD and social determinants of health, as a means toward the elimination of disparate HF outcomes.  [COR 1; C-LD] |
|  | Evidence of health disparities should be monitored and addressed at the clinical practice and the health care system levels.  [COR 1; C-LD] |
|  | **QUALITY METRICS AND REPORTING** |
|  | Performance measures based on professionally developed clinical practice guidelines should be used with the goal of improving quality of care for patients with HF.  [COR 1; B-NR] |
|  | Participation in quality improvement programs, including patient registries that provide bench- mark feedback on nationally endorsed, clinical practice guideline-based quality and performance measures can be beneficial in improving the quality of care for patients with HF.  [COR 2A; B-NR] |
|  | For all patients with HF, palliative and supportive care including high-quality communication, conveyance of prognosis, clarifying goals of care, shared decision-making, symptom management, and caregiver support should be provided to improve QOL and relieve suffering.  [COR 1; C-LD] |
|  | For patients with HF being considered for, or treated with life-extending therapies, the option for discontinuation should be anticipated and discussed through the continuum of care, including at the time of initiation, and reassessed with changing medical conditions and shifting goals of care.  [COR 1; C-LD] |
|  | For patients with HF particularly stage D  HF patients being evaluated for advanced therapies, patients requiring inotropic support or temporary mechanical support, patients experiencing uncontrolled symptoms, major medical decisions, or multimorbidity, frailty, and cognitive impairment—specialist palliative care consultation can be useful to improve QOL and relieve suffering.  [COR 2A; B-R] |

| AF | Atrial Fibrillation | HFrEF | Heart failure with reduced ejection fraction |
| --- | --- | --- | --- |
| ACS | Acute coronary syndrome | ICD | implantable cardioverter-defibrillator |
| ACEi | angiotensin-converting enzyme. | IHD | Ischemic Heart Disease |
| AKUH | Aga Khan University Hospital | LVAD | Left ventricular assist device |
| ARB | Angiotensin receptor blockers | LBBB | Left bundle branch block |
| ARNi | Angiontensin Receptor Neprilysin Inhibitor | LVEF | Left ventricular ejection fraction |
| CT | Computed Tomography | MRI | Magnetic Resonance Imaging |
| CAD | Coronary Artery Disease | MRA | Mineralocorticoid receptors antagonist |
| CCBP | Center for Clinical Best Practices | MI | Myocardial Infraction |
| CHF | Congestive Heart Failure | NYHA | New York Heart Association |
| CPG | Clinical Practice Guidelines | NT-pro BNP | N-terminal prohormone B type natriuretic Peptide |
| CVD | Cardio-vascular Disease | NSAIDs | Non-steroidal anti-inflammatory drugs |
| CPET | Cardiopulmonary Exercise Testing | PCP | Primary Care Physician |
| CMR | Cardiovascular magnetic resonance imaging | PET | Positron Emission Tomography |
| CRTs | Cardiac resynchronization therapies | QoL | Quality of Life |
| DCM | Dilated cardiomyopathy | TTE | Transthoracic Echocardiogram |
| DOAC | Direct Oral AntiCoagulant | TIA | Transient Ischaemic Attack |
| ECG | Electrocardiogram | ToR | Table of Recommendations |
| EF | Ejection Fraction | SCD | Sudden Cardiac Death |
| EtD | Evidence to Decision |  |  |
| GDMT | Guideline directed medical therapy |  |  |
| HF | Heart Failure |  |  |
| HFrEF | Heart failure with reduced ejection fraction |  |  |

**Stable Heart Ischemic Disease**

**Adoloped from:** Guideline for the Diagnosis and Management of Patients with Stable Ischemic Heart Disease

Journal of the American College of Cardiology Vol. 60, No. 24, 2012 © 2012 by the American College of Cardiology Foundation and the American Heart Association, <http://dx.doi.org/10.1016/j.jacc.2012.07.013>

**Adoloped from:** Focused Update of the Guideline for the Diagnosis and Management of Patients with Stable Ischemic Heart Disease**,** Journal of the American College of Cardiology Vol. 64, No. 18, 2014 by the American college of Cardiology Foundation and the American Heart Association, http://dx.doi.org/10.1016/j.jacc.2014.07.017

**Key to the understanding of Class of Recommendations and Levels of Evidence.**

| Classes of recommendation (COR) | | | | | | |
| --- | --- | --- | --- | --- | --- | --- |
| Class I | Class IIa | | Class IIb | Class III | | Class III |
| Strong Benefit >>> Risk | Moderate Benefit >> Risk | | Weak Benefit ≥ Risk | No benefit (Moderate) Benefit = Risk | | Harm (Strong) Risk > Benefit |
| Levels of evidence (LOE) | | | | | | |
| Level A | | Level B | | | Level C | |
| Multiple population evaluated | | Limited population evaluated | | | Very limited population evaluated | |

**Table of Recommendations**

|  | **Diagnosis** |
| --- | --- |
|  | **Clinical Evaluation in the initial diagnosis of SIHD in Patients with Chest pain** |
|  | Patients with chest pain should have a thorough history and physical examination to assess the probability of IHD before additional testing.  [COR I, LOE C] |
|  | Patients who present with acute angina should be categorized as stable or unstable; patients with UA should be further categorized as being at high, moderate, or low risk.  [ COR I, LOE C] |
|  | **Electrocardiography** |
|  | A resting ECG is recommended in patients without an obvious, noncardiac cause of chest pain.  [COR I, LOE B] |
|  | Standard exercise ECG testing is recommended for patients with an intermediate pretest probability of IHD who have an interpretable ECG and at least moderate physical functioning or no disabling comorbidity.  [COR I, LOE A] |
|  | Exercise stress with nuclear MPI or echocardiography is recommended for patients with an intermediate to high pretest probability of IHD who have an uninterpretable ECG and at least moderate physical functioning or no disabling comorbidity.  [COR I, LOE B] |
|  | For patients with a low pre-test probability of obstructive IHD who do require testing, standard exercise ECG testing can be useful, provided the patient has an interpretable ECG and at least moderate physical functioning or no disabling comorbidity.  [COR IIa, LOE C] |
|  | Exercise stress with nuclear MPI or echocardiography is reasonable for patients with an intermediate to high pre-test probability of obstructive IHD who have an interpretable ECG and at least moderate physical functioning or no disabling comorbidity.  [COR IIa, LOE B] |
|  | Pharmacological stress with CMR can be useful for patients with an intermediate to high pre-test probability of obstructive IHD who have an uninterpretable ECG and at least moderate physical functioning or no disabling comorbidity.  [COR IIa, LOE B] |
|  | CCTA might be reasonable for patients with an intermediate pre-test probability of IHD who have at least moderate physical functioning or no disabling comorbidity.  [COR IIb, LOE B] |
|  | For patients with a low pre-test probability of obstructive IHD who do require testing, standard exercise stress echocardiography might be reasonable, provided the patient has an interpretable ECG and at least moderate physical functioning or no disabling comorbidity.  [COR IIb, LOE C] |
|  | Pharmacological stress with nuclear MPI, echocardiography, or CMR is not recommended for patients who have an interpretable ECG and at least moderate physical functioning or no disabling comorbidity.  [COR III: No benefit, LOE C] |
|  | Exercise stress with nuclear MPI is not recommended as an initial test in low-risk patients who have an interpretable ECG and at least moderate physical functioning or no disabling comorbidity.  [COR III: No benefit, LOE C] |
|  | **Unable to Exercise** |
|  | Pharmacological stress with nuclear MPI or echocardiography is recommended for patients with an intermediate to high pre-test probability of IHD who are incapable of at least moderate physical functioning or have disabling comorbidity.  [COR I, LOE B] |
|  | Pharmacological stress echocardiography is reasonable for patients with a low pre-test probability of IHD who require testing and are incapable of at least moderate physical functioning or have disabling comorbidity.  [COR IIa, LOE C] |
|  | CCTA is reasonable for patients with a low to intermediate pre-test probability of IHD who are incapable of at least moderate physical functioning or have disabling comorbidity.  [COR IIa, LOE B] |
|  | Pharmacological stress CMR is reasonable for patients with an intermediate to high pre-test probability of IHD who are incapable of at least moderate physical functioning or have disabling comorbidity.  [B] |
|  | Standard exercise ECG testing is not recommended for patients who have an uninterpretable ECG or are incapable of at least moderate physical functioning or have disabling comorbidity.  [COR III: No benefit, LOE C] |
|  | **Other** |
|  | CCTA is reasonable for patients with an intermediate pre-test probability of IHD who a) have continued symptoms with prior normal test findings, or b) have inconclusive results from prior exercise or pharmacological stress testing, or c) are unable to undergo stress with nuclear MPI or echocardiography.  [COR IIa, LOE C] |
|  | For patients with a low to intermediate pre-test probability of obstructive IHD, non-contrast cardiac CT to determine the CAC score may be considered.  [COR IIb, LOE C] |
|  | **Risk Assessment Advanced testing; resting and stress non-invasive testing resting imaging to a.ssess cardiac structure and function** |
|  |  |
|  | Assessment of resting LV systolic and diastolic ventricular function and evaluation for abnormalities of myocardium, heart valves, or pericardium are recommended with the use of Doppler echocardiography in patients with known or suspected IHD and a prior MI, pathological Q waves, symptoms or signs suggestive of heart failure, complex ventricular arrhythmias, or an undiagnosed heart murmur.  [COR I, LOE B] |
|  | Assessment of cardiac structure and function with resting echocardiography may be considered in patients with hypertension or diabetes mellitus and an abnormal ECG.  [COR IIb, LOE C] |
|  | Measurement of LV function with radionuclide imaging may be considered in patients with a prior MI or pathological Q waves, provided there is no need to evaluate symptoms or signs suggestive of heart failure, complex ventricular arrhythmias, or an undiagnosed heart murmur.  [COR IIb, LOE C] |
|  | Echocardiography, radionuclide imaging, CMR, and cardiac CT are not recommended for routine assessment of LV function in patients with a normal ECG, no history of MI, no symptoms, or signs suggestive of heart failure, and no complex ventricular arrhythmias.  [COR III: No benefit, LOE C] |
|  | Routine reassessment (1 year) of LV function with technologies such as echocardiography radionuclide imaging, CMR, or cardiac CT is not recommended in patients with no change in clinical status and for whom no change in therapy is contemplated.  [COR III: No benefit, LOE C] |
|  | **Stress testing and advanced imaging patients with known SIHD who require non-invasive testing for risk assessment** |
|  | **Able to exercise:** |
|  | Standard exercise ECG testing is recommended for risk assessment in patients with SIHD who are able to exercise to an adequate workload and have an interpretable ECG.  [COR I, LOE B] |
|  | The addition of either nuclear MPI or echocardiography to standard exercise ECG testing is recommended for risk assessment in patients with SIHD who are able to exercise to an adequate workload but have an uninterpretable ECG not due to LBBB or ventricular pacing.  [COR I, LOE B] |
|  | The addition of either nuclear MPI or echocardiography to standard exercise ECG testing is reasonable for risk assessment in patients with SIHD who are able to exercise to an adequate workload and have an interpretable ECG.  [COR IIa, LOE B] |
|  | CCTA may be reasonable for risk assessment in patients with SIHD who are able to exercise to an adequate workload but have an uninterpretable ECG.  [COR IIb, LOE B] |
|  | CMR with pharmacological stress is reasonable for risk assessment in patients with SIHD who are able to exercise to an adequate workload but have an uninterpretable ECG.  [B] |
|  | Pharmacological stress imaging (nuclear MPI, echocardiography, or CMR) or CCTA is not recommended for risk assessment in patients with SIHD who are able to exercise to an adequate workload and have an interpretable ECG.  [COR III: No benefit, LOE C] |
|  | **Unable to Exercise** |
|  | Pharmacological stress with either nuclear MPI or echocardiography is recommended for risk assessment in patients with SIHD who are unable to exercise to an adequate workload regardless of interpretability of ECG.  [COR I, LOE B] |
|  | CCTA can be useful as a first-line test for risk assessment in patients with SIHD who are unable to exercise to an adequate workload regardless of interpretability of ECG.  [COR IIa, LOE C] |
|  | CMR with pharmacological stress is reasonable for risk assessment in patients with SIHD who are able to exercise to an adequate workload but have an uninterpretable ECG  [B] |
|  | **Risk Assessment regardless of the patient’s ability to exercise** |
|  | Pharmacological stress with either nuclear MPI or echocardiography is recommended for risk assessment in patients with SIHD who have LBBB on ECG, regardless of ability to exercise to an adequate workload.  [COR I, LOE B] |
|  | Either exercise or pharmacological stress with imaging (nuclear MPI, echocardiography, or CMR) is recommended for risk assessment in patients with SIHD who are being considered for revascularization of known coronary stenosis of unclear physiological significance.  [COR I, LOE B] |
|  | CCTA can be useful for risk assessment in patients with SIHD who have an indeterminate result from functional testing.  [COR IIa, LOE C] |
|  | CCTA might be considered for risk assessment in patients with SIHD unable to undergo stress imaging or as an alternative to invasive coronary angiography when functional testing indicates a moderate - to high-risk result and knowledge of angiographic coronary anatomy is unknown.  [COR IIb, LOE C] |
|  | A request to perform either a) more than 1 stress imaging study or a stress imaging study and a CCTA at the same time is not recommended for risk assessment in patients with SIHD.  [COR III: No benefit, LOE C] |
|  | **Coronary Angiography as an initial testing strategy to assess risk** |
|  | Patients with SIHD who develop symptoms and signs of heart failure should be evaluated to determine whether coronary angiography should be performed for risk assessment.  [COR I, LOE B] |
|  | **Coronary Angiography to assess risk after initial workup with non-invasive testing** |
|  | Coronary arteriography is recommended for patients with SIHD whose clinical characteristics and results of non-invasive testing indicate a high likelihood of severe IHD and when the benefits are deemed to exceed risk.  [COR I, LOE C] |
|  | Coronary angiography is reasonable to further assess risk in patients with SIHD who have depressed LV function (EF 50%) and moderate risk criteria on noninvasive testing with demonstrable ischemia.  [COR IIa, LOE C] |
|  | Coronary angiography is reasonable to further assess risk in patients with SIHD and inconclusive prognostic information after non-invasive testing or in patients for whom non-invasive testing is contraindicated or inadequate.  [COR IIa, LOE C] |
|  | Coronary angiography for risk assessment is reasonable for patients with SIHD who have unsatisfactory quality of life due to angina, have preserved LV function (EF 50%), and have intermediate risk criteria on non-invasive testing.  [COR IIa, LOE C] |
|  | Coronary angiography for risk assessment is not recommended in patients with SIHD who elect not to undergo revascularization or who are not candidates for revascularization because of comorbidities or individual preferences.  [COR III: No benefit, LOE B] |
|  | Coronary angiography is not recommended to further assess risk in patients with SIHD who have preserved LV function (EF 50%) and low-risk criteria on non-invasive testing.  [COR III: No benefit, LOE B] |
|  | Coronary angiography is not recommended to assess risk in patients who are at low risk according to clinical criteria and who have not undergone non-invasive risk testing.  [COR III: No benefit, LOE C] |
|  | Coronary angiography is not recommended to assess risk in asymptomatic patients with no evidence of ischemia on non-invasive testing.  [COR III: no benefit, LOE C] |
|  | **Treatment and Management** |
|  | **Guideline-directed medical therapy** |
|  | **Lipid management** |
|  | Lifestyle modifications, including daily physical activity and weight management, are strongly recommended for all patients with SIHD.  [COR I, LOE B] |
|  | Dietary therapy for all patients should include reduced intake of saturated fats (to 7% of total calories), trans-fatty acids (to 1% of total calories), and cholesterol (to 200 mg/d).  [COR I, LOE B] |
|  | In addition to therapeutic lifestyle changes, a moderate or high dose of a statin therapy should be prescribed, in the absence of contraindications or documented adverse effects.  [COR I, LOE A] |
|  | For patients who do not tolerate statins, LDL cholesterol–lowering therapy with bile acid sequestrants, niacin, or both is reasonable.  [COR IIa, LOE B] |
|  | **Blood Pressure Management** |
|  | All patients should be counselled about the need for lifestyle modification: weight control; increased physical activity; alcohol moderation; sodium reduction; and emphasis on increased consumption of fresh fruits, vegetables, and low-fat dairy products.  [COR I, LOE B] |
|  | In patients with SIHD with BP 140/90 mm Hg or higher, antihypertensive drug therapy should be instituted in addition to or after a trial of lifestyle modifications.  [COR I, LOE A] |
|  | The specific medications used for treatment of high BP should be based on specific patient characteristics and may include ACE inhibitors and/or beta blockers, with addition of other drugs, such as thiazide diuretics or calcium channel blockers, if needed to achieve a goal BP of less than 140/90 mm Hg.  [COR I, LOE B] |
|  | **Diabetes Management** |
|  | For selected individual patients, such as those with a short duration of diabetes mellitus and a long-life expectancy, a goal hemoglobin A1c (HbA1c) of 7% or less is reasonable.  [COR IIa, LOE B] |
|  | A goal HbA1c between 7% and 9% is reasonable for certain patients according to age, history of hypoglycemia, presence of microvascular or macrovascular complications, or presence of coexisting medical conditions.  [COR IIa, LOE C] |
|  | Initiation of pharmacotherapy interventions to achieve target HbA1c might be reasonable.  [COR IIb, LOE A] |
|  | Therapy with rosiglitazone should not be initiated in patients with SIHD.  [COR III: Harm, LOE C] |
|  | **Physical Activity** |
|  | For all patients, the clinician should encourage 30 to 60 minutes of moderate-intensity aerobic activity, such as brisk walking, at least 5 days and preferably 7 days per week, supplemented by an increase in daily lifestyle activities (e.g., walking breaks at work, gardening, household work) to improve cardiorespiratory fitness and move patients out of the least-fit, least-active, high-risk cohort (bottom 20%).  [COR I, LOE B] |
|  | For all patients, risk assessment with a physical activity history and/or an exercise test is recommended to guide prognosis and prescription.  [COR I, LOE B] |
|  | Medically supervised programs (cardiac rehabilitation) and physician-directed, home-based programs are recommended for at-risk patients at first diagnosis.  [COR I, LOE A] |
|  | It is reasonable for the clinician to recommend complementary resistance training at least 2 days per week.  [COR IIa, LOE C] |
|  | **Weight Management** |
|  | BMI and/or waist circumference should be assessed at every visit, and the clinician should consistently encourage weight maintenance or reduction through an appropriate balance of lifestyle physical activity, structured exercise, caloric intake, and formal behavioral programs when indicated to maintain or achieve a BMI between 18.5 and 24.9 kg/m2 and a waist circumference less than 102 cm (40 inches) in men and less than 88 cm (35 inches) in women (less for certain racial groups).  [COR I, LOE B] |
|  | The initial goal of weight loss therapy should be to reduce body weight by approximately 5% to 10% from baseline. With success, further weight loss can be attempted if indicated.  [COR I, LOE C] |
|  | **Smoking Cessation Counseling** |
|  | Smoking cessation and avoidance of exposure to environmental tobacco smoke at work and home should be encouraged for all patients with SIHD. Follow-up, referral to special programs, and pharmacotherapy are recommended, as is a stepwise strategy for smoking cessation (Ask, Advise, Assess, Assist, Arrange, Avoid).  [COR I, LOE B] |
|  | **Management of Psychological factors** |
|  | It is reasonable to consider screening SIHD patients for depression and to refer or treat when indicated.  [COR IIa, LOE B] |
|  | Treatment of depression has not been shown to improve cardiovascular disease outcomes but might be reasonable for its other clinical benefits.  [COR IIb LOE C] |
|  | **Alcohol Consumption** |
|  | In patients with SIHD who use alcohol, it might be reasonable for nonpregnant women to have 1 drink (4 ounces of wine, 12 ounces of beer, or 1 ounce of spirits) a day and for men to have 1 or 2 drinks a day, unless alcohol is contraindicated (such as in patients with a history of alcohol abuse or dependence or with liver disease).  [COR IIb, LOE C] |
|  | **Avoiding Exposure to Air pollution** |
|  | It is reasonable for patients with SIHD to avoid exposure to increased air pollution to reduce the risk of cardiovascular events.  [COR IIa, LOE C] |
|  | **Additional Medical therapy to prevent MI and Death:** |
|  | **Antiplatelet therapy** |
|  | Treatment with aspirin 75 to 162 mg daily should be continued indefinitely in the absence of contraindications in patients with SIHD.  [COR I, LOE A] |
|  | Treatment with clopidogrel is reasonable when aspirin is contraindicated in patients with SIHD.  [COR I, LOE B] |
|  | Treatment with aspirin 75 to 162 mg daily and clopidogrel 75 mg daily might be reasonable in certain high-risk patients with SIHD.  [COR IIb, LOE B] |
|  | Dipyridamole is not recommended as antiplatelet therapy for patients with SIHD.  [COR III:No benefit, LOE B] |
|  | **Beta- Blocker Therapy** |
|  | Beta-blocker therapy should be started and continued for 3 years in all patients with normal LV function after MI or ACS.  [COR I, LOE B] |
|  | Beta-blocker therapy should be used in all patients with LV systolic dysfunction (EF 40%) with heart failure or prior MI, unless contraindicated. (Use should be limited to carvedilol, metoprolol succinate, or bisoprolol, which have been shown to reduce risk of death.)  [COR I, LOE A] |
|  | Beta blockers may be considered as chronic therapy for all other patients with coronary or other vascular disease.  [COR IIb, LOE C] |
|  | **Renin-Angiotensin-Aldosterone Blocker therapy** |
|  | ACE inhibitors should be prescribed in all patients with SIHD who also have hypertension, diabetes mellitus, LVEF 40% or less, or CKD, unless contraindicated.  [COR I, LOE A] |
|  | ARBs are recommended for patients with SIHD who have hypertension, diabetes mellitus, LV systolic dysfunction, or CKD and have indications for, but are intolerant of, ACE inhibitors.  [COR I, LOE A] |
|  | Treatment with an ACE inhibitor is reasonable in patients with both SIHD and other vascular disease.  [COR IIa, LOE B] |
|  | It is reasonable to use ARBs in other patients who are ACE inhibitor intolerant.  [COR IIa, LOE C] |
|  | **Influenza Vaccination** |
|  | An annual influenza vaccine is recommended for patients with SIHD.  [COR I, LOE B] |
|  | **Additional therapy to reduce risk of MI and death** |
|  | The usefulness of chelation therapy is uncertain for reducing cardiovascular events in patients with SIHD.  [COR IIb, LOE B] |
|  | Estrogen therapy is not recommended in postmenopausal women with SIHD with the intent of reducing cardiovascular risk or improving clinical outcomes.  [COR III: No benefit, LOE A] |
|  | Vitamin C, vitamin E, and beta-carotene supplementation are not recommended with the intent of reducing cardiovascular risk or improving clinical outcomes in patients with SIHD.  [COR III: No benefit, LOE A] |
|  | Treatment of elevated homocysteine with folate or vitamins B6 and B12 is not recommended with the intent of reducing cardiovascular risk or improving clinical outcomes in patients with SIHD.  [COR III: No benefit, LOE A] |
|  | Treatment with garlic, coenzyme Q10, selenium, or chromium is not recommended with the intent of reducing cardiovascular risk or improving clinical outcomes in patients with SIHD.  [COR III:No benefit, LOE C] |
|  | **Medical therapy for relief of symptoms** |
|  | Beta blockers should be prescribed as initial therapy for relief of symptoms in patients with SIHD.  [COR I, LOE B] |
|  | Calcium channel blockers or long-acting nitrates should be prescribed for relief of symptoms when beta blockers are contraindicated or cause unacceptable side effects in patients with SIHD.  [COR I, LOE B] |
|  | Calcium channel blockers or long-acting nitrates, in combination with beta blockers, should be prescribed for relief of symptoms when initial treatment with beta blockers is unsuccessful in patients with SIHD.  [COR I, LOE B] |
|  | Sublingual nitroglycerin or nitroglycerin spray is recommended for immediate relief of angina in patients with SIHD.  [COR I, LOE B] |
|  | CLASS IIa  Treatment with a long-acting non-dihydropyridine calcium channel blocker (verapamil  or diltiazem) instead of a beta blocker as initial therapy for relief of symptoms is reasonable in patients with SIHD.  [COR IIa, LOE B] |
|  | Ranolazine can be useful when prescribed as a substitute for beta blockers for relief of symptoms in patients with SIHD if initial treatment with beta blockers leads to unacceptable side effects or is ineffective or if initial treatment with beta blockers is contraindicated.  [COR IIa, LOE B] |
|  | Ranolazine in combination with beta blockers can be useful when prescribed for relief of symptoms when initial treatment with beta blockers is not successful in patients with SIHD.  [COR IIa, LOE A] |
|  | **Alternative therapies for relief of symptoms in patients with refractory angina** |
|  | Trans myocardial revascularization (TMR) may be considered for relief of refractory angina in patients with SIHD.  [COR IIb, LOE B] |
|  | Acupuncture should not be used for the purpose of improving symptoms or reducing cardiovascular risk in patients with SIHD.  [COR III: No benefit, LOE C] |
|  | **CAD Revascularization** |
|  | **Heart team approach to revascularization decisions** |
|  | A Heart Team approach to revascularization is recommended in patients with unprotected left main or complex CAD.  [COR I, LOE C] |
|  | **Prevention and Screening** |
|  | **Patient Education** |
|  | Patients with SIHD should have an individualized education plan to optimize care and promote wellness, including:  [COR I]  education on the importance of medication adherence for managing symptoms and retarding disease progression.  [LOE C]  An explanation of medication management and cardiovascular risk reduction strategies in a manner that respects the patient’s level of understanding, reading comprehension, and ethnicity. [LOE B]  A comprehensive review of all therapeutic options.  [LOE B]  a description of appropriate levels of exercise, with encouragement to maintain recommended levels of daily physical activity.  [LOE C]  introduction to self-monitoring skills.  [LOE C]  Information on how to recognize worsening cardiovascular symptoms and take appropriate action.  [LOE C] |
|  | Patients with SIHD should be educated about the following lifestyle elements that could influence prognosis: weight control, maintenance of a BMI of 18.5 to 24.9 kg/m2 , and maintenance of a waist circumference less than 102 cm (40 inches) in men and less than 88 cm (35 inches) in women (less for certain racial groups); lipid management; BP control; smoking cessation and avoidance of exposure to second-hand smoke; and individualized medical, nutrition, and lifestyle changes for patients with diabetes mellitus to supplement diabetes treatment goals and education.  [COR I, LOE C] |
|  | It is reasonable to educate patients with SIHD about; [COR IIa]   1. Adherence to a diet that is low in saturated fat, cholesterol, and trans-fat; high in fresh fruits, whole grains, and vegetables; and reduced in sodium intake, with cultural and ethnic preferences incorporated.   [LOE B]   1. Common symptoms of stress and depression to minimize stress related angina symptoms.   [LOE C]   1. Comprehensive behavioral approaches for the management of stress and depression.   [LOE C]   1. Evaluation and treatment of major depressive disorder when indicated.   [LOE B] |
|  | **Follow up** |
|  | **Patient Follow-Up: Monitoring of Symptoms and Antianginal Therapy** |
|  | **Clinical Evaluation, Echocardiography during routine periodic follow-up** |
|  | Patients with SIHD should receive periodic follow-up, at least annually, that includes all of the following:  Assessment of symptoms and clinical function;  Surveillance for complications of SIHD, including heart failure and arrhythmias;  Monitoring of cardiac risk factors; and  Assessment of the adequacy of and adherence to recommended lifestyle changes and medical therapy.  [COR I, LOE C] |
|  | Assessment of LVEF and segmental wall motion by echocardiography or radionuclide imaging is recommended in patients with new or worsening heart failure or evidence of intervening MI by history or ECG.  [COR I, LOE C]  Periodic screening for important comorbidities that are prevalent in patients with SIHD, including diabetes mellitus, depression, and CKD, might be reasonable.  [COR IIb, LOE C] |
|  | A resting 12-lead ECG at 1-year or longer intervals between studies in patients with stable symptoms might be reasonable.  [COR IIb, LOE C] |
|  | Measurement of LV function with a technology such as echocardiography or radionuclide imaging is not recommended for routine periodic reassessment of patients who have not had a change in clinical status or who are at low risk of adverse cardiovascular events.  [COR III:No benefit, LOE C] |
|  | **Non-invasive testing in known SIHD** |
|  | **Patients able to Exercise** |
|  | Standard exercise ECG testing is recommended in patients with known SIHD who have new or worsening symptoms not consistent with UA and who have a) at least moderate physical functioning and no disabling comorbidity and b) an interpretable ECG.  [COR I, LOE B] |
|  | Exercise with nuclear MPI or echocardiography is recommended in patients with known SIHD who have new or worsening symptoms not consistent with UA and who have at least moderate physical functioning or no disabling comorbidity but  an uninterpretable ECG.  [COR I, LOE B] |
|  | Exercise with nuclear MPI or echocardiography is reasonable in patients with known SIHD who have new or worsening symptoms not consistent with UA and who have  at least moderate physical functioning and no disabling comorbidity, previously required imaging with exercise stress, or known multivessel disease or high risk for multivessel disease.  [COR IIa, LOE B] |
|  | Pharmacological stress imaging with nuclear MPI, echocardiography, or CMR is not recommended in patients with known SIHD who have new or worsening symptoms not consistent with UA and who are capable of at least moderate physical functioning or have no disabling comorbidity.  [COR III: No benefit, LOE C] |
|  | **Patients unable to Exercise** |
|  | Pharmacological stress imaging with nuclear MPI or echocardiography is recommended in patients with known SIHD who have new or worsening symptoms not consistent with UA and who are incapable of at least moderate physical functioning or have disabling comorbidity.  [COR I, LOE B] |
|  | Standard exercise ECG testing should not be performed in patients with known SIHD who have new or worsening symptoms not consistent with UA and who a) are incapable of at least moder- ate physical functioning or have disabling comorbidity or b) have an uninterpretable ECG.  [COR III: No benefit, LOE C] |
|  | **Irrespective of Ability to Exercise** |
|  | CCTA for assessment of patency of CABG or of coronary stents 3 mm or larger in diameter might be reasonable in patients with known SIHD who have new or worsening symptoms not consistent with UA, irrespective of ability to exercise.  [COR IIb, LOE B] |
|  | CCTA might be reasonable in patients with known SIHD who have new or worsening symptoms not consistent with UA, irrespective of ability to exercise, in the absence of known moderate or severe calcification or if the CCTA is intended to assess coronary stents less than 3 mm in diameter.  [COR IIb, LOE B] |
|  | CCTA should not be performed for assessment of native coronary arteries with known moderate or severe calcification or with coronary stents less than 3 mm in diameter in patients with known SIHD who have new or worsening symptoms not consistent with UA, irrespective of ability to exercise.  [COR III: No benefit, LOE B] |
|  | **Non-invasive testing in known SIHD – Asymptomatic (or stable symptoms)** |
|  | Nuclear MPI, echocardiography, or CMR with either exercise or pharmacological stress can be useful for follow-up assessment at 2-year or longer intervals in patients with SIHD with prior evidence of silent ischemia or who are at high risk for a recurrent cardiac event and; are unable to exercise to an adequate workload, have an uninterpretable ECG, or have a history of incomplete coronary revascularization.  [COR IIa, LOE C] |
|  | Standard exercise ECG testing performed at 1-year or longer intervals might be considered for follow-up assessment in patients with SIHD who have had prior evidence of silent ischemia or are at high risk for a recurrent cardiac event and are able to exercise to an adequate workload and have an interpretable ECG.  [COR IIb, LOE C] |
|  | In patients who have no new or worsening symptoms or no prior evidence of silent ischemia and are not at high risk for a recurrent cardiac event, the usefulness of annual surveillance exercise ECG testing is not well established.  [COR IIb, LOE C] |
|  | Nuclear MPI, echocardiography, or CMR, with either exercise or pharmacological stress or CCTA, is not recommended for follow-up assessment in patients with SIHD, if performed more frequently than at a) 5-year intervals after CABG or b) 2-year intervals after PCI.  [COR III: No benefit LOE C] |

**ACRONYMS & ABBREVIATIONS**

| ACE | Angiotensin-Converting Enzyme | IHD | Ischemic Heart Disease |
| --- | --- | --- | --- |
| AV | Atrioventricular | LV | Left Ventricular |
| ARB | Angiotensin Receptor Blockers | LDL | Low-Density Lipoprotein |
| BMI | Body Mass Index | LBBB | Left bundle-branch block |
| CAC | Coronary Artery Calcium | LVEF | Left ventricular ejection fraction |
| CAD | Coronary Artery Disease | MI | Myocardial infarction |
| CKD | Chronic Kidney Disease | MPI | Myocardial Perfusion Imaging |
| CMR | Cardiovascular Magnetic Resonance Imaging | PAD | Peripheral Artery Disease |
| CCTA | Coronary Computed Tomography Angiography | PCI | Percutaneous Coronary Intervention |
| CABG | Coronary Artery Bypass Grafting. | SIHD | Stable Ischemic Heart Disease |
| ECG | Electrocardiogram | TMR | Transmyocardial Revascularization |
| EF | Ejection Fraction | UA | Unstable Angina |
